# Genomes of four *Streptomyces* strains reveal insights into putative new species and pathogenicity of scab-causing organisms

**DOI:** 10.1186/s12864-023-09190-y

**Published:** 2023-03-23

**Authors:** Laura Henao, Ramin Shirali Hossein Zade, Silvia Restrepo, Johana Husserl, Thomas Abeel

**Affiliations:** 1 Department of Civil and Environmental Engineering, Universidad de los Andes, 111711 Bogotá, Colombia; 2 Delft Bioinformatics Lab, Delft University of Technology, 2628 XE Delft, Netherlands; 3 Laboratory of Mycology and Phytopathology - (LAMFU), Department of Chemical and Food Engineering, Universidad de los Andes, 111711 Bogotá, Colombia; 4 Infectious Disease and Microbiome Program, Broad Institute of MIT and Harvard, 415 Main Street, Cambridge, MA 02142 USA

**Keywords:** *Streptomyces*, Horizontal transfer, Scab-causing species, Potential virulence factors, Secondary metabolites, Putative biosynthetic clusters

## Abstract

Genomes of four *Streptomyces* isolates, two putative new species (*Streptomyces* sp. JH14 and *Streptomyces* sp. JH34) and two non thaxtomin-producing pathogens (*Streptomyces* sp. JH002 and *Streptomyces* sp. JH010) isolated from potato fields in Colombia were selected to investigate their taxonomic classification, their pathogenicity, and the production of unique secondary metabolites of Streptomycetes inhabiting potato crops in this region. The average nucleotide identity (ANI) value calculated between *Streptomyces* sp. JH34 and its closest relatives (92.23%) classified this isolate as a new species. However, *Streptomyces* sp. JH14 could not be classified as a new species due to the lack of genomic data of closely related strains. Phylogenetic analysis based on 231 single-copy core genes, confirmed that the two pathogenic isolates (*Streptomyces* sp. JH010 and JH002) belong to *Streptomyces pratensis* and *Streptomyces xiamenensis*, respectively, are distant from the most well-known pathogenic species, and belong to two different lineages. We did not find orthogroups of protein-coding genes characteristic of scab-causing Streptomycetes shared by all known pathogenic species. Most genes involved in biosynthesis of known virulence factors are not present in the scab-causing isolates (*Streptomyces* sp. JH002 and *Streptomyces* sp. JH010). However, Tat-system substrates likely involved in pathogenicity in *Streptomyces* sp. JH002 and *Streptomyces* sp. JH010 were identified. Lastly, the presence of a putative mono-ADP-ribosyl transferase, homologous to the virulence factor scabin, was confirmed in *Streptomyces* sp. JH002. The described pathogenic isolates likely produce virulence factors uncommon in *Streptomyces* species, including a histidine phosphatase and a metalloprotease potentially produced by *Streptomyces* sp. JH002, and a pectinesterase, potentially produced by *Streptomyces* sp. JH010. Biosynthetic gene clusters (BGCs) showed the presence of clusters associated with the synthesis of medicinal compounds and BGCs potentially linked to pathogenicity in *Streptomyces* sp. JH010 and JH002. Interestingly, BGCs that have not been previously reported were also found. Our findings suggest that the four isolates produce novel secondary metabolites and metabolites with medicinal properties.

**Supplementary Information:**

The online version contains supplementary material available at 10.1186/s12864-023-09190-y.

## Introduction

In general, *Streptomyces* species are characterized by the production of interesting secondary metabolites; many of them are used for the treatment of a wide range of diseases. Therefore *Streptomyces* spp. are often considered a primary source of drug compounds [1, 2, 3]. In the environment, these metabolites may increase the fitness of *Streptomyces* spp. [4]. These natural compounds are involved in nutrient or niche competition, mutualism, and symbiotic relationships between the microorganisms and plants or insects [4, 5, 6].

Under laboratory culture conditions, however, *Streptomycetes* often only produce a small part of the secondary metabolites they can synthesize [7]. The discovery of metabolites by traditional methods requires the detection of these compounds in culturable conditions, reducing the chances of finding novel metabolites [2, 7]. Biosynthetic gene cluster (BGC) evaluation by genome mining and bioinformatics enables the identification and characterization of metabolites that cannot be found otherwise through traditional methods [7]. BGCs encoding secondary metabolites diverge between species and even strains [5, 8], likely due to acquisition through horizontal gene transfer or deletion [5]. These differences in BGCs often lead to adaptation of these microorganisms to the ecosystem, inducing lineage divergence by subsequent niche differentiation or antagonism [5]. Since BGCs are highly diverse at the strain level, even genome mining of strains belonging to the same species are key for the discovery of novel secondary metabolites [9].

Most *Streptomyces* species are saprophyte organisms and few have been described as plant pathogens [1, 10]. Pathogenic *Streptomyces* spp. are not host-specific and can infect potato tubers and taproot crops producing scab disease [11]. In these crops, pathogenic *Streptomyces* deteriorate tuber and root vegetable appearance decreasing their commercial value and causing high economic losses worldwide [12, 13]. Pathogenic *Streptomyces* species use different strategies to infect plants and to cause scab disease, including phytotoxic secondary metabolites, phytohormones, and secreted proteins [10].

Most studies aimed at understanding virulence mechanisms in scab-causing species have focused on strains that produce thaxtomin phytotoxins, including *Streptomyces scabiei* 87–22, *Streptomyces scabiei* EF-35, *Streptomyces europaeiscabiei* 89–04, *Streptomyces acidiscabies* 84–104, *Streptomyces stelliscabiei* NRRL B-24447, and *Streptomyces turgidiscabies* Car8. Among the virulence factors also identified in these pathogens are cytokinins, scabin, indole-3-acetic acid, concanamycins, coronafacoyl phytotoxins, Nec1 protein, TomA, ethylene, and suberinases [10, 14, 15, 16, 17, 18, 19, 20, 21, 22, 23]. In contrast, little is known about the infection mechanisms employed by non-thaxtomin producing *Streptomyces* species. Virulence factors of *Streptomyces luridiscabiei, Streptomyces puniciscabiei*, *Streptomyces niveiscabiei,* and *Streptomyces reticuliscabiei* have not been stablished so far. Few virulence factors have been described for some non-thaxtomin producing pathogens, including Fridamycin E, FD-891, Borrelidin and non-diketopiperazine; however, their role in disease development remains unclear [24, 25, 26, 27].

Recently, several *Streptomyces* isolates from potato crops in Colombia were characterized [28]. The authors identified several scab-causing isolates that did not produce thaxtomin A. Virulence factors responsible for the pathogenic phenotype in these organisms were not identified. Within these isolates, *Streptomyces* sp. JH002 and *Streptomyces* sp. JH010, were identified as *Streptomyces xiamenensis* and *Streptomyces pratensis,* respectively*,* based on a multilocus sequence analysis (MLSA). In addition, two isolates (*Streptomyces* sp. JH14 and *Streptomyces* sp. JH34) could not be classified into specific taxa and were considered potentially new species [28]. From inoculation of sporulated isolates on potato tuber slices and radish seedling bioassays, *Streptomyces* sp. JH002 and *Streptomyces* sp. JH010 were classified as pathogens [28]. However, the pathogenicity of *Streptomyces* sp. JH14 and *Streptomyces* sp. JH34 could not be established, as these microorganisms did not sporulate on ISP2 or oatmeal agar [28] or GYM (glucose, yeast and malt extract) agar.

In this study we wanted to establish the taxonomic classification of *Streptomyces* sp. JH14, *Streptomyces* sp. JH34, *Streptomyces* sp. JH010 and *Streptomyces* sp. JH002 and investigate if JH14 and JH34 could be new species based on genomic data. In addition, we wanted to evaluate the pathogenic isolates to find potential virulence factors produced by these strains. Finally, we wanted to search for putative BGCs in the genomes of the four isolates, looking for potentially interesting metabolites. Our results highlight the importance of focusing scab disease research on non-thaxtomin-producing scab-causing species to provide new insights into the emergence of novel pathogenic *Streptomyces* species. In addition, our results contribute to the study of the diversity of Streptomycetes and may lead to the discovery of new medicinal compounds.

## Results

### Genome characterization of four *Streptomyces* spp

The genome assemblies of the isolates were nearly complete, with more than 98% of the single copy orthologs from the actinobacteria_odb9 BUSCO database represented and between 1 and 2 contigs representing each genome (Table 1). The genome sizes of *Streptomyces* sp. JH002, *Streptomyces* sp. JH34, *Streptomyces* sp. JH010, and *Streptomyces* sp. JH14 isolates ranged between 6.24 Mbp and 7.72 Mbp. *Streptomyces* sp. JH010 had the largest genome (7.72 Mbp). The genomic GC content of *Streptomyces* sp. JH002, *Streptomyces* sp. JH010, *Streptomyces* sp. JH34, and *Streptomyces* sp. JH14 isolates, ranged from 70.2% to 72%. All genome assemblies and annotations are available at Genbank under Genebank identifiers CP087989, JAJSOO000000000, JAJNMN000000000, and JAJNEL000000000, for JH002, JH34, JH010, and JH14, respectively. Raw sequencing data has been submitted to NCBI with BioProject PRJNA657491.

**Table 1:** Results of assembly, annotation, and completeness analysis for the genomes of *Streptomyces* sp. JH002, *Streptomyces* sp. JH34, *Streptomyces* sp. JH010 and *Streptomyces* sp. JH14

Isolate	JH002	JH34	JH010	JH14
**Assembly**				
Number of contigs	1	2	2	2
Contigs N50 (bp)	6,242,747	7,279,370	7,656,094	6,580,952
Assembled genome size (bp)	6,242,747	7,292,977	7,718,394	6,928,808
Coverage (X)	530	562	507	479
G + C (%)	72	70.9	71	70.2
**BUSCO** (actinobacteria_odb9)				
Complete and single copy	348	351	351	351
Complete and duplicate	0	0	0	0
Fragmented	2	0	0	0
Missing	2	1	1	1
Total BUSCO genes	352	352	352	352
BUSCO completeness (%)	98.9	99.7	99.7	99.7
**Annotation**				
Number of CDS	5,676	6,606	6,883	6,294
Hypothetical gene annotations (%)	31	34	33	33
CDSs classified into a subsystem (%)	35	34	34	35
Number of RNAs	72	88	85	85
Number of rRNAs (16S, 23S, 5S)	15	18	18	21
**Genbank identifier**	CP087989	JAJSOO000000000	JAJNMN000000000	JAJNEL000000000

The number of coding sequences (CDSs) predicted by the RAST server in the four isolates ranged from 5,676 to 6,883. About 34%-35% of CDSs found in the *Streptomyces* genomes were classified into a subsystem by the RAST server. Isolates showed to be very different among each other in terms of their metabolism (Table 2). There were important differences between the number of CDSs associated with each subsystem in the different isolates. Within the four isolates, *Streptomyces* sp. JH14 has the highest number of CDSs in the “Phages, Prophages, Transposable elements, Plasmids” subsystem (14 CDSs), “Cofactors, Vitamins, Prosthetic Groups, Pigments” (335 CDSs) and “Metabolism of Aromatic Compounds” (86 CDSs). *Streptomyces* sp. JH010 has the highest number of CDSs in the “iron acquisition and metabolism” subsystem (64 CDSs) and “stress response” (180 CDSs). In the latter subsystem, *Streptomyces* sp. JH34 also has a high number of CDSs (171 CDSs). In contrast, *Streptomyces* sp. JH14 has the lowest number of CDSs associated with “Dormancy and Sporulation” (2 CDSs), and no CDSs were classified into “Secondary metabolism”. *Streptomyces* sp. JH002 contains the highest number of CDSs linked to “Secondary metabolism” (27 CDSs) and “Virulence, disease and defense” (68 CDSs) subsystems.

**Table 2:** Number of genes of *Streptomyces* sp. JH002, *Streptomyces* sp. JH34, *Streptomyces* sp. JH010, and *Streptomyces* sp. JH14 isolates distributed by subsystem based on RAST annotation server

Subsystem	Number of genes			
	***Streptomyces* sp. JH002**	***Streptomyces* sp. JH34**	***Streptomyces* sp. JH010**	***Streptomyces* sp. JH14**
Motility and Chemotaxis	5	7	5	6
Phages, Prophages, Transposable elements, Plasmids	6	6	6	14
Dormancy and Sporulation	6	12	12	2
Potassium metabolism	16	18	18	23
Sulfur Metabolism	25	63	56	72
Secondary Metabolism	27	9	4	0
Miscellaneous	31	42	39	45
Nitrogen Metabolism	33	27	30	29
Metabolism of Aromatic Compounds	34	60	54	86
Cell Division and Cell Cycle	35	34	37	34
Phosphorus Metabolism	42	38	43	50
Iron acquisition and metabolism	52	50	64	30
Regulation and Cell signaling	59	63	65	57
Virulence, Disease and Defense	68	46	43	55
Membrane Transport	101	113	109	96
RNA Metabolism	103	119	117	105
Cell Wall and Capsule	107	138	123	158
DNA Metabolism	110	124	130	115
Nucleosides and Nucleotides	113	122	133	124
Respiration	117	129	126	151
Stress Response	127	171	180	138
Fatty Acids, Lipids, and Isoprenoids	240	258	248	248
Cofactors, Vitamins, Prosthetic Groups, Pigments	258	287	269	335
Protein Metabolism	290	306	309	283
Carbohydrates	476	564	563	488
Amino Acids and Derivatives	500	591	644	553
Total number of genes	2981	3397	3427	3297

### Taxonomic analysis suggests that JH34 and JH14 are putative new species

Pairwise comparison results between 16S rRNA sequences from *Streptomyces* sp. JH34 and EZBioCloud database showed 30 species with similarity values ≥ 98.7% (Table S2), including two species with 100% of similarity (*Streptomyces clavifer* CGMCC 4.1604 and *Streptomyces mutomycini* NRRL B-65393). The 16S rRNA gene from *Streptomyces* sp. JH14 had a similarity ≥ 98.7% to 36 species (Table S3). Within these species, *Streptomyces yanii* NBRC 14,669 had the highest 16S rRNA gene similarity value (99.9%).

Although the genomic sequences of all the species with 16S rRNA gene similarity values ≥ 98.7% are supposed to be included for the calculation of ANI calculations [29], the genomes of several of these species are not available. Therefore, an MLSA was conducted to evaluate which species within the available genome sequences were closest to *Streptomyces* sp. JH34 and *Streptomyces* sp. JH14. The closest species to the isolates were selected for ANI calculation.

**Fig. 1:**
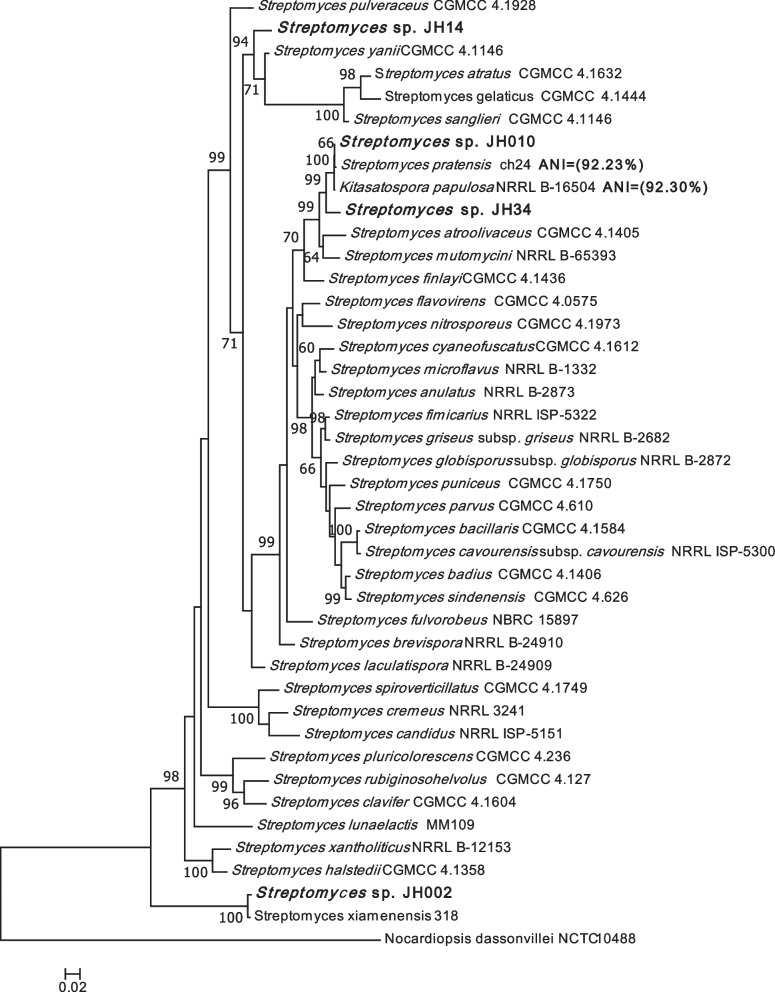
Phylogenetic analysis based on concatenated sequences of *atpD*, *gyrB, recA, rpoB* and *trpB* genes of the *Streptomyces* sp. JH34, *Streptomyces* sp. JH14, *Streptomyces* sp. JH002, and *Streptomyces* sp. JH010 and 37 *Streptomyces* reference strains. Phylogenetic tree was constructed using the ML method. *Nocardiopsis dassonvillei* NCTC 10,488 was chosen as the outgroup. The data were resampled 1000 times for Bootstrap test. Only bootstrap values higher than 60% are shown. As previously described [28] *Streptomyces* sp. JH10 and *Streptomyces* sp. JH002 belong to *S. pratensis* and *S. xiamenensis* species. ANI values between *Streptomyces* sp. JH34 and S. pratensis and ‘*K. papulosa’* (closest relatives) are shown in parentheses. ANI value between JH14 and *S. yanii* (closest relative) could not be calculated as its genome has not been sequenced

MLSA placed the isolate *Streptomyces* sp. JH34 in a well-supported clade (Bootstrap value = 99%) along with *Streptomyces pratensis* ch24, ‘*Kitasatospora papulosa’* NRRL B-16504 (considered as a member of *S. pratensis* [30]), *Streptomyces atroolivaceus* CGMCC 4.1405*,* and *Streptomyces mutomycini* NRRL B-65393 species, being more closely related to *S. pratensis* and ‘*K. papulosa’* (Fig. 1). MLSA results differed from the 16S rRNA similarity analysis, which indicated that *Streptomyces* sp. JH34 was most closely related to *S. clavifer* and *S. mutomycini*. Nevertheless, the ANI values confirmed MLSA results, being higher between *Streptomyces* sp. JH34 and *S. pratensis* ATCC 33,331 (92.23%) and ‘*K. papulosa’* (92.30%), than between *Streptomyces* sp. JH34 and *S. mutomycini* (89.24%), *S. atroolivaceus* (89.15%), and *S. clavifer* (86.18%). The ANI values between *Streptomyces* sp. JH34 and its closest relatives are lower than 95%, indicating that this isolate is a new species.

MLSA grouped the isolate *Streptomyces* sp. JH14 with *Streptomyces yanii* CGMCC 4.1146, *Streptomyces sanglieri* CGMCC 4.1146, *Streptomyces gelaticus* CGMCC 4.1444 and *Streptomyces atratus* CGMCC 4.1632 in a well-supported clade (Bootstrap = 92%), with the three latter species distantly related from *S. yanni* and *Streptomyces* sp. JH14 (Fig. 1). *Streptomyces yanni* was selected for ANI calculation as it is the closes relative to JH14 based on MLSA. However, it could not be calculated as the genome of *S. yanii* has not been sequenced. Consequently, even though some results suggest that *Streptomyces sp.* JH14 could be a new species, *S. yanni’s* full genomic information is needed to confirm this hypothesis based on genomic data.

### Scab causing *Streptomyces* sp. JH010 and *Streptomyces* sp. JH002 are phylogenetically distant from other phytopathogenic *Streptomyces* species

**Fig. 2:**
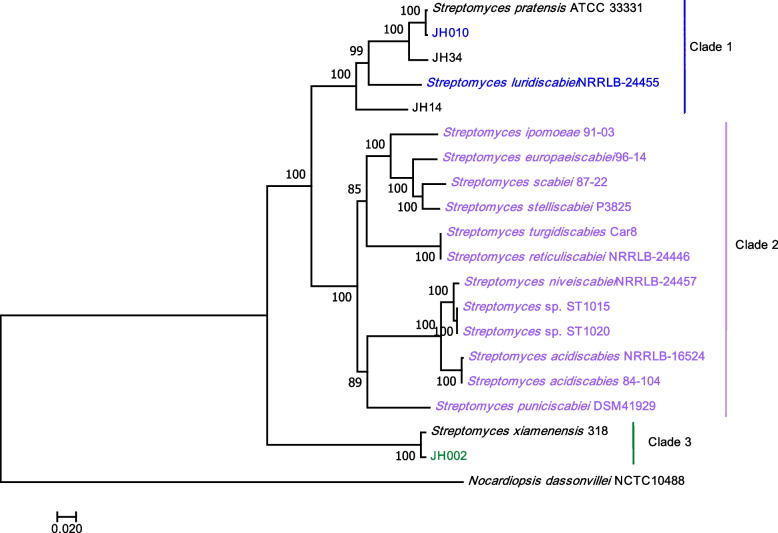
Phylogenetic analysis of *Streptomyces* species based on concatenated sequences of 231 single-copy core genes of isolates *Streptomyces* sp. JH002, *Streptomyces* sp. JH010, *Streptomyces* sp. JH14, *Streptomyces* sp. JH34, and 13 known pathogenic *Streptomyces* species. Pathogenic organisms are highlighted in the colors blue, purple, and green. Also, the type strains *S. pratensis* ATCC 33,331 and *S. xiamenensis* 318 were included in this analysis. The phylogenetic tree was constructed using the ML method. *Nocardiopsis dassonvillei* NCTC 10,488 was chosen as the outgroup. Data were resampled 1000 times for bootstrapping

Our data show that *Streptomyces* sp. JH002 and *Streptomyces* sp. JH010 are distantly related to most of the scab-causing species (Fig. 2). A phylogenetic analysis based on concatenated sequences of 231 single-copy core genes from pathogenic isolates *Streptomyces* sp. JH010 and *Streptomyces* sp. JH002 and well-known pathogenic *Streptomyces* species showed that these isolates belong to two further different lineages. The isolates *Streptomyces* sp. JH002 and *Streptomyces* sp. JH010 were grouped with the non-pathogenic species, *S. xiamenensis,* and *S. pratensis* (Bootstrap value = 100%). Pathogenic *Streptomyces* spp. were mainly clustered in three well-supported clades (Bootstrap values = 100%), two of them constituted by previously described pathogenic species (Clade 1 and Clade 2) (Fig. 2). Most of the well-known *Streptomyces* pathogenic species are placed in clade 2, including *S. scabiei*, *S. acidiscabies*, *S. europaeiscabiei,* and *S. turgidiscabieis*. This clade groups all thaxtomin-producing species; however, it also contains species that do not produce this type of toxins (i.e., *Streptomyces reticuliscabiei*, *Streptomyces* sp. ST1015, and *Streptomyces* sp. ST1020). This clade is distantly related to clades 1 and 3. Clade 1 was constituted by two pathogens*, Streptomyces* sp. JH010 and *S. luridiscabiei* NRRL B-24455, and clade 3 contained only one pathogen, *Streptomyces* sp. JH002. This clade is the most distant clade from the well-known pathogenic *Streptomyces* species.

### Biosynthetic gene clusters

Using antiSMASH, we found several putative biosynthetic gene clusters in the genomes of *Streptomyces* sp. JH002, *Streptomyces* sp. JH34, *Streptomyces* sp. JH010, and *Streptomyces* sp. JH14. The four isolates contain BGCs associated with the production of secondary metabolites with antimicrobial and antitumoral activities and iron chelators used for the treatment of different diseases (Table 3). In addition, we found BGCs probably linked to the synthesis of novel natural compounds; in the genomes of the pathogenic isolates, we also found several BGCs that might be related to the pathogenesis of these organisms, including the BGC for ectoine, melanin, and several siderophores (i.e., Desferrioxamin B/E and coelichelin).

**Table 3:** Putative biosynthetic gene clusters found by antiSMASH from the genomes of *Streptomyces* sp. JH002, *Streptomyces* sp. JH34, *Streptomyces* sp. JH010, and *Streptomyces* sp. JH14. Only clusters with similarity (percentage of genes with significant BLAST hit) ≥ 60% are shown. Sm (%) = similarity percentage. NA = Metabolite function not fully established, or without therapeutic activity

Isolate	Cluster name	Sm (%)	Secondary metabolite function
***Streptomyces* sp. JH002**	Ikarugamycin	84	Antimicrobial activity
	Ectoine	100	NA
	Moomysin	75	NA
	Nenestatin	66	NA
	Desferrioxamine B	60	Iron chelator
***Streptomyces* sp. JH34**	Isorenieratene	100	NA
	Desferrioxamine B/ E	100	Iron chelator
	Ectoine	100	NA
	Melanin	100	NA
	Sceliphrolactam	92	Antifungal metabolite
	Coelichelin	90	Iron chelator
	Spore pigment	83	NA
	Chromomycin A3	88	Antitumoral metabolite
***Streptomyces* sp. JH010**	Melanin	100	NA
	Ectoine	100	NA
	Ectoine	100	NA
	Isorenieratene	100	NA
	Coelichelin	90	Iron chelator
	Sceliphrolactam	88	Antifungal metabolite
	Spore pigment	83	NA
	Desferrioxamine B /E	83	Iron chelator
	Terpene	69	NA
	Carbapenem MM4550	65	Antimicrobial activity
***Streptomyces* sp. JH14**	Desferrioxamine B	100	Iron chelator
	Naringenin	100	Antimicrobial, anti-inflammatory, and antitumoral metabolite
	Amycomicin	100	Antimicrobial metabolite
	Hopene	84	NA

The genomes of *Streptomyces* sp. JH002, *Streptomyces* sp. JH34, *Streptomyces* sp. JH010, and *Streptomyces* sp. JH14 contained 23, 27, 27, and 11 BGCs for secondary metabolites, respectively, based on antiSMASH annotation (Tables S6-S9). The BGCs predicted by antiSMASH comprised genes classified in several subsystems by RAST annotation server, including “secondary metabolism”, “stress response”, “iron acquisition and metabolism”, “dormancy and sporulation”, and “virulence, disease and defense” subsystems. Only between 22 and 36% of these clusters had similarity values ≥ 60% to known biosynthetic clusters.

*Streptomyces* sp. JH14 contains putative BGCs to produce the antibiotic amycomicin, flavanone naringenin and desferrioxamin B. *Streptomyces* sp. JH34 contains BGCs like those involved in the production of Chromomycin A_3_, desferrioxamin B, and sceliphrolactam. In *Streptomyces* sp. JH010 we also found a BGC associated with the production of sceliphrolactam. No differences were found between the clusters identified by antiSMASH in the genomes of *Streptomyces* sp. JH010 and *Streptomyces pratensis* ATCCC 33,331. The genome of *Streptomyces* sp. JH010 contains putative BGCs for ectoine and melanin, metabolites that may be associated with the pathogenicity of this isolate.

*Streptomyces* sp. JH002 has a gene cluster similar to the one for the production of the antibiotic ikarugamycin. The genome of *Streptomyces* sp. JH002 also contains four clusters that are not present in *Streptomyces xiamenensis* 318. Most of the clusters annotated in this genome had low percentages of similarity to known biosynthesis gene arrays (10%-75%). In JH002 we also found ectoine and desferrioxamin B BGCs that may be involved in the pathogenicity/virulence of this isolate.

### Virulence factors in *Streptomyces* sp. JH010 and *Streptomyces* sp. JH002

**Fig. 3:**
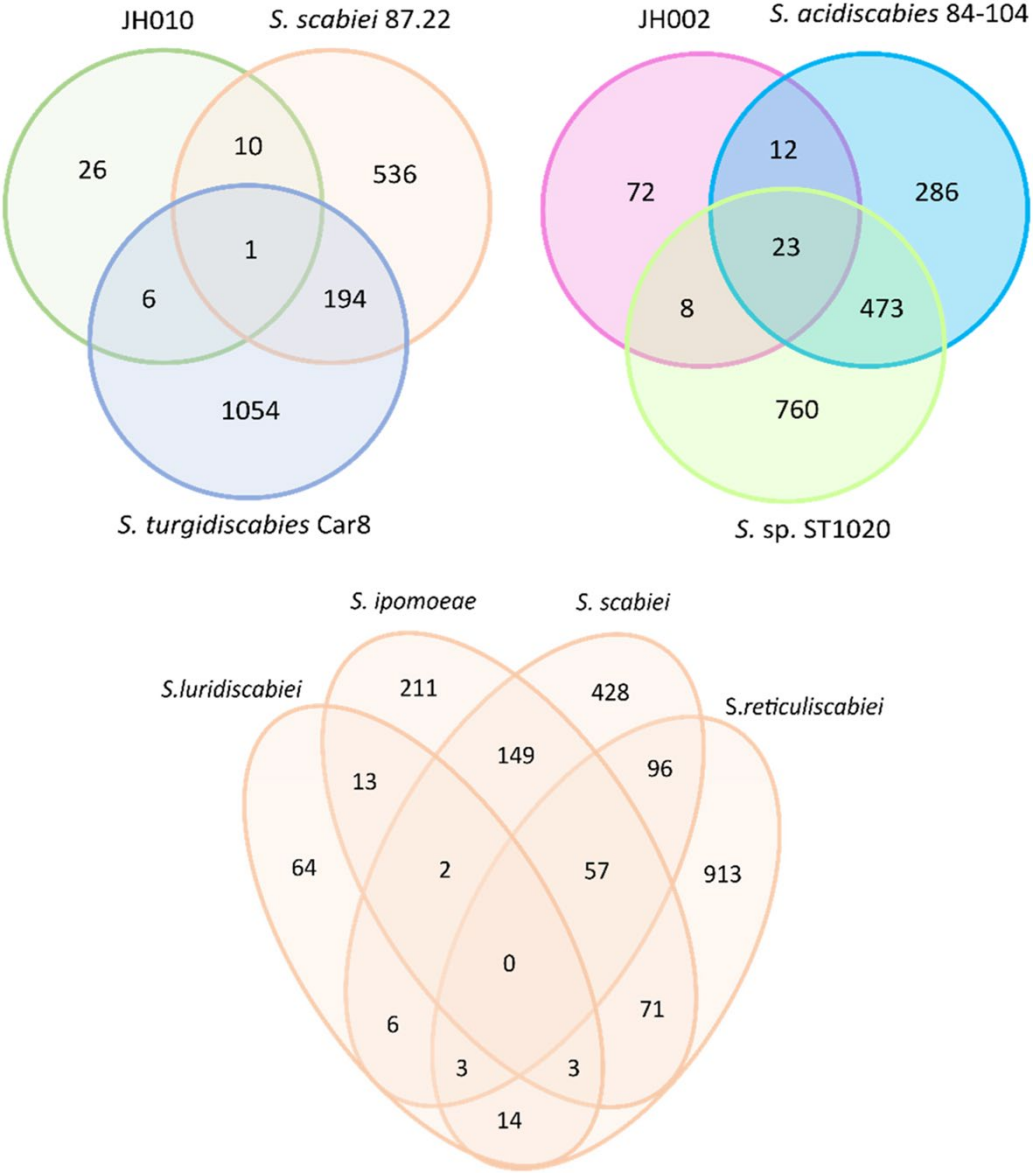
Venn diagram for orthogroups of protein-coding genes unique in pathogenic *Streptomyces* species

Orthologous gene analysis results revealed that *Streptomyces* species do not share unique orthologous gene clusters characteristic of pathogenic organisms (Fig. 3). Furthermore, BlastP search showed that most virulence factors identified in the pathogenic *Streptomyces* species are not present in the genomes of *Streptomyces* sp. JH002 or *Streptomyces* sp. JH010.

Orthofinder assigned 192,325 genes, 94.2% of the identified genes, into 15,607 orthogroups. In total, 43 and 115 of the orthogroups identified in *Streptomyces* sp. JH010 and *Streptomyces* sp. JH002, respectively, were specific for pathogenic *Streptomyces* species. *Streptomyces* sp. JH010 shared most orthogroups with *S. scabiei* 87.22 (11) and *S. turgidiscabies* Car8 (7), and *Streptomyces* sp. JH002 shared most orthogroups with *S. acidiscabies* (35) and *Streptomyces* sp. ST1020 (31) (Fig. 3).

In *Streptomyces* sp. JH010, the orthogroups shared with other pathogens did not contain homologous genes implied in the pathogenicity/virulence of phytopathogenic organisms. In contrast, in JH002, we found two orthogroups for genes encoding proteins associated with the virulence of plant pathogenic bacteria, including a histidine phosphatase, and a metalloprotease [31–33].

Key proteins for the production of thaxtomins (thaxtomin synthases A and B) are not encoded in the isolates’ genomes (Tables 4 and 5). Homologs to proteins required for the synthesis of other phytotoxins associated with the pathogenesis of scab-causing *Streptomyces* species were also not found. Within the proteins recognized as potential virulence factors in the *Streptomyces* genus, only a homolog of scabin was found in *Streptomyces* sp. JH002. In addition, homologs of the IAM hydrolase (*iaaH* gene) required for IAA production in the indole-3-acetamide pathway were found in both pathogenic isolates *Streptomyces* sp. JH010 and *Streptomyces* sp. JH002; however, a Trp monooxygenase-like protein, necessary for IAA production in this pathway, was not found. The twin-arginine translocation (Tat) system was also found encoded in the genomes of both isolates; we found TatA, TatB, and TatC homologs.

**Table 4:** Blastp results. Proteins encoded in the genome of *Streptomyces* sp. JH002 with significant alignments to proteins involved in virulence mechanisms in pathogenic *Streptomyces* species are shown. Protein family was identified for each sequence using the HMMER server. Protein sequences with query cover ≥ 80% are shown in bold

Name	Reference protein		Putative Protein encode in *Streptomyces sp.* JH002 genome					
	**Protein Family**	**Length**	**Protein ID**	**Protein family**	**Length**	**Query Cover (%)**	**Positive (%)**	**Identity (%)**
Trp monooxygenase	Flavin-containing amine oxidoreductase	565	PRJNA657491:H7827_04665	Flavin-containing amine oxidoreductase	470	23	41	31
			PRJNA657491:H7827_23025	Flavin-containing amine oxidoreductase	433	10	60	48
IAM hydrolase	Carbon–nitrogen hydrolase	262	PRJNA657491:H7827_03825	Carbon–nitrogen hydrolase	282	63	49	36
			**PRJNA657491:H7827_06430**	**Carbon–nitrogen hydrolase**	**267**	**93**	**48**	**32**
scabin	-	208	PRJNA657491:H7827_06625	**-**	**197**	**80**	**77**	**62**
TatA	mttA/Hcf106 family	97	PRJNA657491:H7827_02105	mttA/Hcf106 family	86	50	89	53
			**PRJNA657491:H7827_26400**	**mttA/Hcf106 family**	**101**	**96**	**68**	**49**
TatB	mttA/Hcf106 family	168	**PRJNA657491:H7827_09295**	**mttA/Hcf106 family**	**157**	**100**	**83**	**78**
TatC	Sec-independent protein translocase protein (TatC)	317	**PRJNA657491:H7827_26405**	**Sec-independent protein translocase protein (TatC)**	**299**	**100**	**67**	**52**

**Table 5:** Blastp results. Proteins encoded in the genome of *Streptomyces* sp. JH010 with significant alignments to proteins involved in virulence mechanisms in pathogenic *Streptomyces* species are shown. Protein family was identified for each sequence using the HMMER server. Protein sequences with query cover ≥ 80% are shown in bold

**Protein**	**Reference protein**		**Putative Protein encode in *Streptomyces* sp. JH10 genome**					
	**Protein Family**	**Length**	**Protein ID**	**Protein family**	**Length**	**Query Cover (%)**	**Positive (%)**	**Identity (%)**
Trp monooxygenase	Flavin-containing amine oxidoreductase	565	PRJNA657491:H8R03_26010	Flavin-containing amine oxidoreductase	495	**0**	**0**	**0**
			PRJNA657491:H8R03_31270	Flavin containing amine oxidoreductase	421	7	66	42
IAM hydrolase	Carbon–nitrogen hydrolase	262	**PRJNA657491:H8R03_27235**	**Carbon–nitrogen hydrolase**	**280**	**100**	**46**	**31**
			**PRJNA657491:H8R03_30185**	**Carbon–nitrogen hydrolase**	**292**	**87**	**46**	**38**
			**PRJNA657491:H8R03_05035**	**Carbon–nitrogen hydrolase**	**265**	**100**	**83**	**74**
			**PRJNA657491:H8R03_16235**	**Carbon–nitrogen hydrolase**	**265**	**98**	**47**	**33**
TatA	mttA/Hcf106 family	97	PRJNA657491:H8R03_29530	mttA/Hcf106 family	79	57	69	55
			**PRJNA657491:H8R03_06065**	**mttA/Hcf106 family**	**97**	**96**	**75**	**65**
			**PRJNA657491:H8R03_04485**	**mttA/Hcf106 family**	**90**	**85**	**61**	**45**
TatB	mttA/Hcf106 family	168	**PRJNA657491:H8R03_04500**	**mttA/Hcf106 family**	**147**	**100**	**54**	42
			**PRJNA657491:H8R03_21560**	**mttA/Hcf106 family**	**162**	**100**	**64**	**53**
TatC	Sec-independent protein translocase protein (TatC)	317	**PRJNA657491:H8R03_06060**	**Sec-independent protein translocase protein (TatC)**	**319**	**100**	**72**	**58**

#### Tat-system and its effect on virulence

Homologs to TatA, TatB, and TatC, involved in the twin-arginine translocation (Tat) system, were found in the genomes of *Streptomyces* sp. JH002 and *Streptomyces* sp. JH010. To evaluate if any Tat-transported substrates might be associated with the pathogenicity/virulence in *Streptomyces* sp. JH002 and *Streptomyces* sp. JH010, we found several *bona fide* Tat-substrates using TATFIND 1.4 and TatP 1.0 servers.

Forty-two and sixty putative proteins secreted by the Tat-system were found in *Streptomyces* sp. JH002 and *Streptomyces* sp. JH010, respectively, including several plant cell wall degrading enzymes (Table S12). In *Streptomyces* sp. JH002 and *Streptomyces* sp. JH010 we found a putative endo-1,4-beta-xylanase A precursor and a putative endo-1,4-beta-xylanase, respectively. A putative aldose 1-epimerase was also found in both isolates. In *Streptomyces* sp. JH010 we also found three putative enzymes involved in the breakdown of plant cell wall, including several glycosyl hydrolases, and a pectinesterase [34, 35]. In *Streptomyces* sp. JH002 we found a putative rhamnogalacturonan lyase. In this strain we also found a lon-like protease, and a peptidase containing the S8/S53 domain.

#### Scabin homolog in Streptomyces sp. JH002

**Fig. 4:**
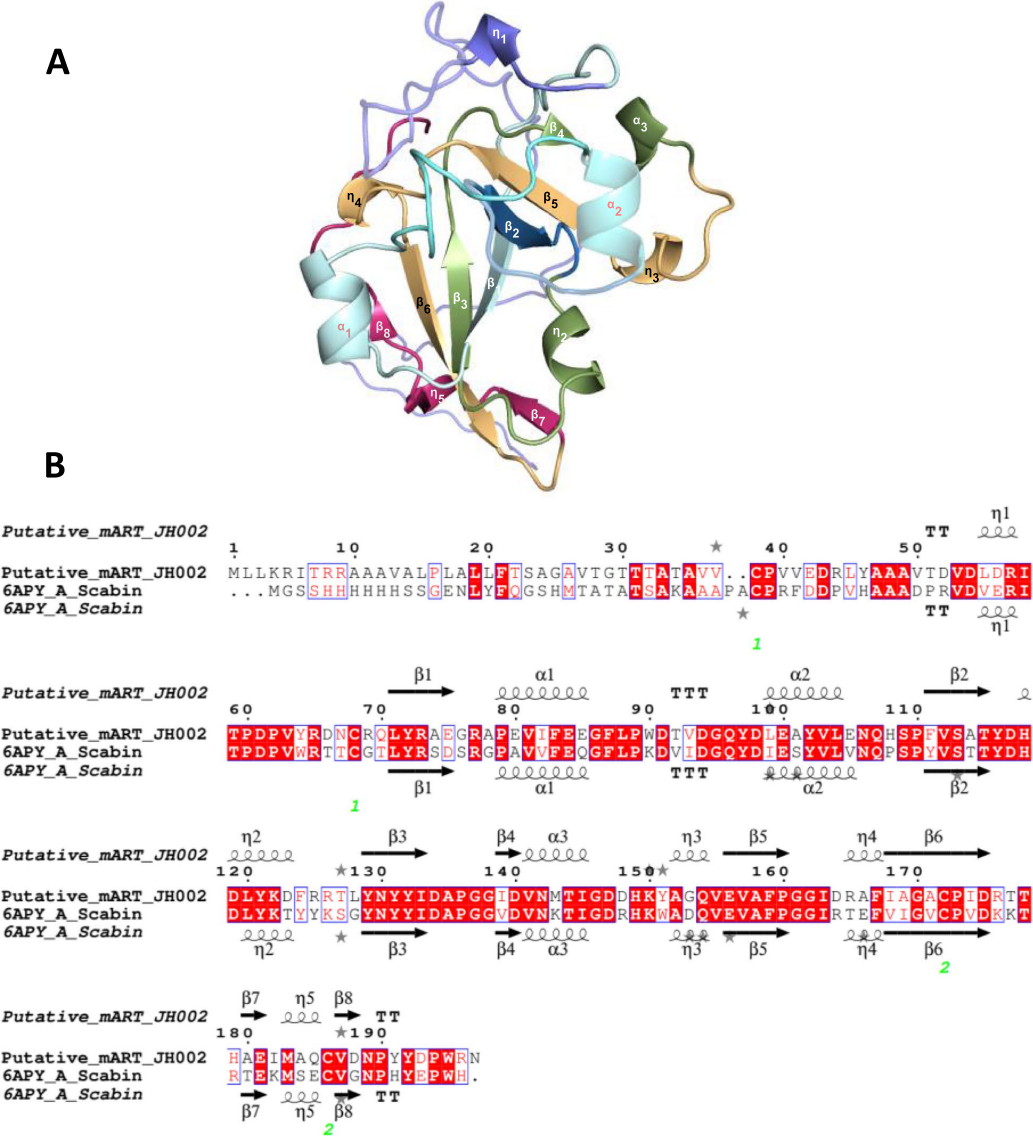
**A** 3D structure predicted in RaptorX from putative mART sequence. The image was obtained by using Chimera 1.15rc. **B** Alignment of putative mART-JH002 and Scabin sequences. Secondary structures of each protein are also shown. The image was obtained through ESPript 3.0 server under default parameters [37]. Identical residues are highlighted in red and similar residues are in blue frames. β strands are shown as arrows, α helices as squiggles, strict β-turns as TT and strict α-turns as TTT

In the genome of *Streptomyces* sp. JH002, we found a scabin homolog (mART-JH002), which can be involved in pathogenicity. The prediction of the 3D structure of the mART-JH002, through LOMETS and RaptorX servers, revealed that this protein can be folded into a shape like other mART toxins. Results generated by LOMETS showed that 10 out of 10 servers predicted the crystal structure of scabin as the best 3D model for mART-JH002 with a coverage of 77–83%. Furthermore, the structure predicted by RaptorX showed that the putative 3D structure of mART-JH002 is similar to scabin (Fig. 4). The quality scores of the predicted structure indicated that it has a correct fold (*p*-value = 1.21E-10, Global Distance Test normalized (uGDT) = 148, and the number of identical residues in the alignment normalized (SeqID) = 50%). Moreover, SignalP 5.0 and SecretomeP 2.0 scores (SignalP 5.0 Likelihood = 0.8342; SecP score = 0.591) higher than 0.45, indicated that mART-JH002 is not associated with the bacterial membrane or cell wall and that mART-JH002 might be secreted following the signal peptide pathway [36].

Two out of three key active sites characteristic of mARTs are conserved in mART-JH002, including the Arg required for NAD^+^ binding and the Gln-X-Glu motif necessary for transferase activity (Figure S1). The third active site, commonly constituted by the Ser-Thr-[Ser-Gln-Thr] motif and involved in the scaffold of the NAD^+^ binding pocket formation [18], is replaced by Ser-Ala-Thr- motif in mART-JH002. Despite of the substitution of threonine by an alanine in this active site, this protein can still have DNA ADP-ribosyltransferase activity according to the molecular function predicted by COFACTOR server (Cscore = 0.83).

**Fig. 5:**
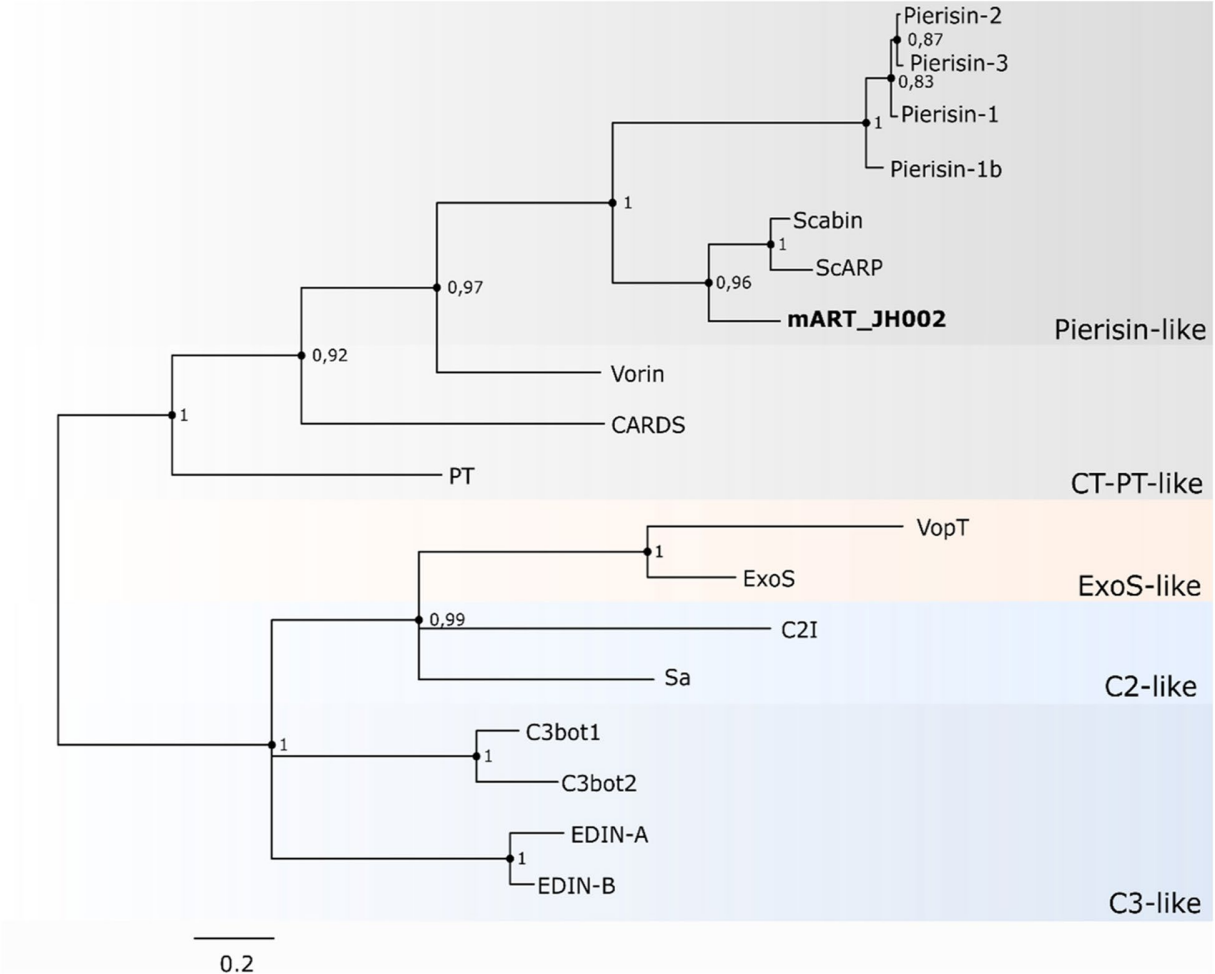
Phylogenetic tree of mARTs and putative mARTs. Tree was constructed by using Bayesian inference

The phylogenetic analysis showed that mART-JH002 is closely related to the Pierisin-like protein family with a high Bayesian support value (Posterior probability = 1.0). Within this group of mARTs, mART-JH002 was relatively more closely related to mARTs from other *Streptomyces* species (scabin and ScARP) than to those described in other organisms (Fig. 5). However, mART-JH002 was distantly related to scabin and ScARP.

## Discussion

The genome sizes of *Streptomyces* sp. JH002, *Streptomyces* sp. JH34, *Streptomyces* sp. JH010 are consistent with the sizes of the genomes of other *Streptomyces* strains previously reported (ranging from 5.93 Mbp to 10.13 Mbp) [38, 39]. The genome of *Streptomyces* sp. JH010, which was identified as *Streptomyces pratensis,* is consistent with those described in other *Streptomyces pratensis* strains (7.52–7.62 Mbp) [40]. Intraspecies genome size variations might be associated with the number of duplicate genes present in each organism. In fact, a positive correlation between the number of paralogues and genome size in several *Streptomyces* species has been previously reported [39]. The genomic GC content of *Streptomyces* sp. JH002, *Streptomyces* sp. JH010, *Streptomyces* sp. JH34, and *Streptomyces* sp. JH14 is consistent with the GC content reported for other *Streptomyces* species, usually over 70% [38, 39]. The number of coding sequences (CDSs) predicted by the RAST is consistent with CDS reported in other *Streptomyces* species (5,491–8,396) and is positively correlated with genome size, a hallmark of bacterial genomes [38, 39].

The phylogenetic relationships observed in this study were consistent with a previous MLSA constructed from sequences of more than 600 *Streptomyces* strains [30]. The fact that *Streptomyces* sp. JH002 and *Streptomyces* sp. JH010 were grouped with the non-pathogenic species, *S. xiamenensis,* and *S. pratensis* with a Bootstrap value of 100%, confirms the previous taxonomic classification [28].

Based on our genome-based taxonomic analysis, we have good evidence that *Streptomyces* sp. JH34 is a putative new species and *Streptomyces* sp. JH14 is likely a candidate to be a putative new species. However, some species with high similarity values based on the 16S rRNA sequence to *Streptomyces* sp. JH34 and *Streptomyces* sp. JH14 were not included in the MLSA, as their genome sequences are not available. These species are *Streptomyces sundarbansensis* MS1/7 closely related to *Streptomyces* sp. JH34 and *Streptomyces* sp. JH14 with 16S rRNA pairwise similarity of 98.96% and 98.89%, respectively, and *Pilimelia columellifera* subsp. *pallida,* closely related to *Streptomyces* sp. JH14 with 98.89% of 16S rRNA pairwise similarity. Also, the genomic sequence of *Streptomyces yanni* i*s* needed for ANI calculation to evaluate if *Streptomyces* sp. JH14 is in fact a new species.

Although the hypothesis that JH14 and JH34 are new species cannot be confirmed based on the available genomic information alone, the phenotypic differences between these strains and their closest relatives make this hypothesis stronger. A previous characterization of *Streptomyces* sp. JH34 and *Streptomyces* sp. JH14 showed that these microorganisms do not sporulate in oatmeal agar or ISP2 medium [28]. However, the *Streptomyces* species most closely related to *Streptomyces* sp. JH34 and *Streptomyces* sp. JH14 can sporulate on these culture media. *Streptomyces pratensis*, and *S. atroolivaceus* produce spores on both oatmeal agar and ISP2, and *S. mutomycini* on oatmeal agar [41, 42]. Likewise, *Streptomyces yanni* can sporulate on oatmeal agar, *Streptomyces gelaticus* sporulates on ISP2 agar and *Streptomyces gelaticus* in both media [42, 43]. In addition, we compared the phylogenetic relationships obtained from the single-copy core genes and MLSA. Both phylogenetic analyses were consistent showing that *Streptomyces* sp. JH34 and *S. pratensis* are closely related and share a common ancestor. These results encourage the hypothesis that *Streptomyces* sp. JH34 is a new species.

It is interesting to find that Scab causing *Streptomyces* sp. JH010 and *Streptomyces* sp. JH002 are not closely phylogenetically related to other scab causing *Streptomyces* spp. In fact, most of the potential virulence factors found in *Streptomyces* sp. JH010 and *Streptomyces* sp. JH002 are different from those reported for other scab-causing *Streptomyces* spp. Based on PCR analysis, it was previously reported that genes involved in the synthesis of thaxtomins and the Nec1 protein, common in pathogenic *Streptomyces* species [10], were not found in *Streptomyces* sp. JH010 and *Streptomyces* sp. JH002 [28]. The absence of genes related to the production of virulence factors described in most pathogenic *Streptomyces* species in *Streptomyces* sp. JH002 and *Streptomyces* sp. JH010, as well as evidence that there are no shared orthogroups specific for scab between all well-known pathogenic organisms and *Streptomyces* sp. JH002 and *Streptomyces* sp. JH010, suggest that the later *Streptomyces* species have gone through different evolutionary paths leading to the pathogenic phenotype. These species may have evolved mainly through horizontal transfer events. Indeed, horizontal transfer has been described as a key process in the evolution of plant pathogenic bacteria, leading to the adaptation of the bacteria to the host [44, 45]. The acquisition of mobile elements (i.e., phages, and integrative and conjugative elements) has enabled the adaptation of phytopathogens to a specific host and has been associated with the development of different symptoms in plants [45].

*Streptomyces* sp. JH010 and *Streptomyces* sp. JH002 contain interesting gene clusters associated with potential virulence factors. In particular, the genome of *Streptomyces* sp. JH010 contains putative BGCs for ectoine and melanin, metabolites that may be associated with the pathogenicity of this isolate. Ectoine and melanin protect pathogens from environmental changes generated during plant infection. Ectoine helps bacteria resist in environments with high osmolarity [46]. Melanin also plays an important role in the survival of microorganisms under adverse environmental conditions and has been implicated broadly in bacterial pathogenesis [47]. *Streptomyces* sp. JH010 also possesses gene arrays similar to siderophore BGCs (i.e., Desferrioxamin B/E and coelichelin). Siderophores in *Streptomyces* species trigger diverse biological processes, including growth, cellular differentiation, and the production of antibiotics [48]. These metabolites have also been associated with the pathogenic phenotype of several plant pathogenic bacteria [49]. Histidine phosphatases, which are potentially produced by JH 002, have been described as virulence regulators of *Xanthomonas campestris* pv. *campestris* [33]. Metalloproteases, also potentially produced by this strain, have been characterized in several pathogenic bacteria, including *Pectobacterium carotovorum*, *Dickeya dadantii*, and *Xanthomonas campestris* [32]. Although the role of these proteins in pathogenicity has not been fully elucidated, it has been proposed that histidine phosphatases might be involved in the breakdown of the plant cell wall or/and helping to counter the plant’s immune response [32].

The Tat-system secretes different proteins associated with virulence in *Streptomyces scabiei* [50] and it was previously established that *Δtatc Streptomyces scabiei* (clone in which the gene *tatc* was mutated*)* was less virulent than the wild type. The Tat-system is known to be involved in virulence in *Streptomyces scabiei* [50] through the secretion of various proteins. We found that the twin-arginine translocation system is encoded in the genomes of both isolates, *Streptomyces* sp. JH010 and *Streptomyces* sp. JH002; we also found several interesting putative Tat substrates in the pathogenic isolates. Together, these two findings suggest that the Tat-system may be involved in the pathogenicity/virulence of these organisms, yet further experimental analyses are required to better evaluate this hypothesis. Of the over hundred putative proteins secreted by the Tat-system in JH002 and JH010, there were several plant cell wall degrading enzymes, which are frequently used in phytopathogenic organisms to make the host susceptible to infection and release nutrients during plant colonization [35, 51]. Endo-1,4-beta-xylanases A and endo-1,4-beta-xylanases, similar to those found in JH002 and JH010, respectively, are involved in xylan degradation, which is a structural polymer found in plant cells [52]. An aldose 1-epimerase from *Phytophthora* species, similar to those potentially produced by JH002 and JH010, has been showed to trigger cell death in *Nicotiana benthamiama* [53]. Other enzymes found in JH010, annotated as glycosyl hydrolases, and a pectinesterase, could be involved in the breakdown of plant cell wall [34, 35]. Rhamnogalacturonan lyases, similar to the one potentially produced by JH002, degrade rhamnogalacturonan I, a structural component of pectin in the cell wall of plants [54, 55]. Lon-like proteases, also potentially produced by this strain, are generally required in pathogenic bacteria for full virulence (i.e., *Pseudomonas syringae* and *Rhizobium radiobacter*) [32]. It was previously reported that the disruption of a gene encoding a protein belonging to the S8 peptidase protein family produced a decrease in the virulence of the fungal pathogen *Penicillium expansum* on apple fruit [56].

In the genome of *Streptomyces* sp. JH002, we found a scabin homolog (mART-JH002). Scabin is a mono-ADP-ribosyltranferase (mART) belonging to the Pierisin family [57]. These enzymes transfer an ADP ribose to DNA [58]. Pierisin-like toxins can induce cell apoptosis by labeling a guanine base with an ADP-ribose moiety [58, 59]. Although the role of scabin in the pathogenicity of *S. scabiei* has not been fully elucidated, it has been observed that scabin modifies DNA and shows a high affinity for the DNA of *Solanum tuberosum* [60], suggesting this scabin homolog may also play an important role in pathogenicity in JH002. Considering that mARTs conserve the reaction mechanism and that mARTs are classified according to substrate target type [36], the divergence of mART-JH002 from other *Streptomyces* pierisin-like enzymes indicate that the targets of this protein might vary from those described for scabin (double or single stranded DNA) and ScARP (mononucleotides and nucleosides) [18, 61].

As expected, we found that all isolates contain gene clusters associated with production of interesting compounds, yet only few of the BCGs we found, had high similarity values to known clusters, suggesting the potential for identification of several novel metabolites in these isolates. Flavanone naringenin, one of the substances potentially produced by *Streptomyces* sp. JH14, has diverse therapeutic properties, including antimicrobial, anti-inflammatory, and antitumor activities [62, 63]; desferrioxamin B, slso potentially produced by this strain, is a siderophore used to treat iron overdose in humans [64]. Chromomycin A_3,_ a metabolite potentially produced by *Streptomyces* sp. JH34 is an antitumoral substance [65] and sceliphrolactam, also potentially produced by this strain is an antifungal [66]. JH002 does not have the region that contains the genes associated with the production of xiamenmycin, an anti-fibrotic drug candidate known to be produced by *S. xiamenensis* 318 [39].

All isolates contain other interesting putative BGCs involved in the production of medicinal substances, including known antimicrobial, anti-inflammatory, and anti-tumoral metabolites; further analysis of these isolates, for example using metabolomic tools, could lead to the identification and isolation of novel compounds or compounds with medicinal and industrial uses.

## Conclusions

*Streptomyces* spp. are very diverse, and there are still many unknowns regarding their pathogenicity and their capacity to produce medicinal substances. Especially in some countries in Latin America, known for their biodiversity, little is known about *Streptomyces* spp. Four strains of *Streptomyces* spp. previously isolated from potato fields in Colombia, were investigated in this study. Based on genomic data, and considering phenotypic differences with closest relatives, we were able to establish that *Streptomyces* sp. JH34 is likely new species. *Streptomyces* sp. JH14 could not be classified as a new species from ANI calculation, because its closest relative has not been sequenced; however, MSLA and phenotypic characteristics suggest it could be a new species as well. We confirmed previous findings that *Streptomyces* sp. JH002 and *Streptomyces* sp. JH010 belong to *Streptomyces pratensis* and *Streptomyces xiamenensis,* respectively*,* and that they are phylogenetically distant from the most well-known pathogenic species. In fact, no orthogroups of protein-coding genes characteristic of scab-causing Streptomycetes were found in the pathogenic isolates and most of the genes involved in the biosynthesis of known virulence factors were also not found in these scab-causing isolates. However, we did find several Tat-system substrates that are probably involved in the pathogenicity of *Streptomyces* sp. JH002 and *Streptomyces* sp. JH010 as well as the presence of a putative mono-ADP-ribosyl transferase, a homolog to scabin, in *Streptomyces* sp. JH002.

We found BGCs for secondary metabolites associated with pathogenicity in the pathogenic isolates (*Streptomyces* sp. JH010 and *Streptomyces* sp. JH002) and BGCs associated with the synthesis of interesting medicinal compounds, including antibiotics, antifungal, and antitumoral substances, in all isolates. Our results provide new insights about pathogenicity in *Streptomyces* species, highlighting the importance of focusing scab disease research on non-thaxtomin-producing scab-causing species and highlights the key role of horizontal transfer in the emergence of new scab-causing organisms. Our results may also contribute to the discovery of new therapeutic agents.

## Methods

### Microbial isolates

The *Streptomyces* species analyzed (*Streptomyces* sp. JH34, *Streptomyces* sp. 14, *Streptomyces* sp. JH010 and *Streptomyces* sp. JH002) were isolated in the department of Cundinamarca, Colombia, from potato tubers. The isolates were phenotypically characterized in a previous study and are deposited at the Museo de Historia Natural ANDES [28].

### DNA isolation

Cultures of isolates *Streptomyces* sp. JH34, *Streptomyces* sp. 14, *Streptomyces* sp. JH010 and *Streptomyces* sp. JH002 were grown in 100 mL ISP2 broth ((Dextrose (4 g/L); Yeast Extract (4 g/L); Malt Extract (10 g/L); pH 7.0—7.2) [67]) for 5 days at 30 °C in constant shaking (250 rpm). After growth, cultures were centrifuged at 11,000 × *g* for 15 min. The supernatant was carefully removed, and *Streptomyces* mycelia were recovered and used for DNA isolation using the DNeasy PowerSoil Kit following the manufacturer’s protocol with the following modifications: i) approximately 0.20 g of mycelium sample was added to the PowerBead Tube instead of a soil sample; ii) three mycelia samples for each isolate were processed separately up until the addition of solution C4, a highly concentrated salt solution used in the DNA isolation in the PowerSoil Kit. Then, the three samples were loaded into the same MB spin column. Washing and elution steps were carried out according to the manufacturer’s protocol.

### Genome sequencing, assembly, and annotation

We sequenced, assembled, and annotated the genomes of four Streptomyces isolates (two pathogens and two putative new species) isolated from potato fields in Colombia. Whole-genome sequencing of the four isolates (*Streptomyces* sp. JH002, *Streptomyces* sp. JH34, *Streptomyces* sp. JH010, and *Streptomyces* sp. JH14) was carried out at the University of Minnesota Genomics Center using Single-Molecule Real-Time (SMRT) Pacific BioSciences (PacBio) technology. Samples were sequenced in one Sequel SMRT Cell 1 M v3. Demultiplexed data was provided and used for de novo assembly of the genomes of *Streptomyces* sp. JH002, *Streptomyces* sp. JH34, *Streptomyces* sp. JH010, and *Streptomyces* sp. JH14; for this, we used the Flye assembler 2.6 [68] using plasmid flag and three polishing iterations; the remaining parameters were set to default. Genome assembly completeness was analyzed by assessing the presence of single-copy ortholog genes using BUSCO 3.01 [69]. The genome sequences obtained were compared to Actinobacteria genes from the OrthoDB database (actinobacteria_odb9). After assembly and BUSCO analyses, we annotated the four genomes on the RAST 2.0 annotation server by the ClassicRAST scheme [70] using default parameters. Finally, Barrnap v.0.9 (https://github.com/tseemann/barrnap) was used to determine the number of rRNAs in each genome.

### Taxonomic classification of *Streptomyces *sp. JH34 and *Streptomyces* sp. JH14 isolates from genome data

Taxonomic classification of *Streptomyces* sp. JH34 and *Streptomyces* sp. JH14 was conducted from the calculation of the Average Nucleotide Identity (ANI) between the isolates and their closest relatives, because ANI differentiates closely related species based on a comparison of genome sequences [29]. To identify the species close to *Streptomyces* sp. JH34 and *Streptomyces* sp. JH14, similarity values between 16S rRNA sequences of the isolates and 16S rRNA sequences available on the EZBioCloud 16S database (https://help.ezbiocloud.net/ezbiocloud-16s-database/) were obtained by pairwise comparison [29]. Species with similarity values ≥ 98.7% are chosen for ANI calculation [29]. Here, we identified the closest species to *Streptomyces* sp. JH34 and *Streptomyces* sp. JH14, based on Multilocus Sequence Analysis (MLSA) of the concatenated sequences of five housekeeping genes (*atpD*, *gyrB*, *recA*, *rpoB*, *trpB*). MLSA has shown a high resolution in the differentiation of close *Streptomyces* species [30]. All the species with 16S rRNA similarity values ≥ 98.7% [29] were selected for the MLSA. The gene sequences for the isolates were obtained from genome assemblies, and the sequences for the reference *Streptomyces* species were retrieved from the NCBI database. The homologous sequences for each housekeeping gene were aligned by using Multiple Sequence Alignment (MUSCLE) [71] and trimmed manually to the same position by using MEGA7 [72]. The resulting alignments were joined head-to-tail in a frame, obtaining 2532 bp sequences, including gaps. Subsequently, the phylogenetic tree was constructed using the Maximum Likelihood (ML) method and the GRT + G + I substitution model in MEGA7 [72]. Pairwise distances were calculated under default parameters. The confidence of the phylogenetic tree and the pairwise distance calculation was estimated by bootstrapping method, resampling the sequences 1000 times. In total, 36 species were included in MLSA, and *Norcadopsis dassonvillei* NCTC 10,488 was chosen as the outgroup. Genbank accession numbers of housekeeping genes for all strains included in MLSA are shown in Table S1. After conducting the MLSA, the closest species to *Streptomyces* sp. JH34 and *Streptomyces* sp. JH14 were chosen based on the phylogenetic analysis results. ANI values between the chosen species and *Streptomyces* sp. JH34 and *Streptomyces* sp. JH14 were obtained by using the ANI Calculator on the EZBioCloud platform [73]. Accession numbers of *Streptomyce*s species assemblies used for ANI calculation are shown in Table S3.

### Phylogenetic analysis

Phylogenetic analysis was conducted based on concatenated sequences of 231 single-copy core genes of the pathogenic isolates *Streptomyces* sp. JH002 and *Streptomyces* sp. JH010, their closest relatives (*S. pratensis* and *S. xiamenensis*), and previously reported pathogenic *Streptomyces* species. The set of single-copy core genes was selected by comparison of the gene identifiers obtained after RAST annotation. Sequences of the homologous genes were aligned using MUSCLE, and the alignments were cleaned by G-block implementation to improve the phylogenetic reconstruction [74]. Subsequently, phylogenetic tree topology was constructed based on aligned sequences using the Maximum Likelihood method with the RAxML program on CIPRES Science Gateway [75, 76]. The data was resampled 1000 times for bootstrap analyses, and the GRTGAMMA model was used as the substitution model. The *Streptomyces* species included in the analysis and the accession numbers of genome assemblies are shown in Table S5.

### Search for putative biosynthetic gene clusters (BGCs)

Biosynthetic gene clusters for secondary metabolites encoded in the genomes of the four isolates (*Streptomyces* sp. JH14, *Streptomyces* sp. JH34, *Streptomyces* sp. JH010, and Streptomyces sp. JH002), *S. pratensis* ATCC 3333, and *S. xiamenensis* 318 were identified using the antiSMASH 5.0 online tool [77]. The two latter strains are considered saprophytic bacteria; however, they are the closest phylogenetically related strains to the pathogenic isolates (JH002 and JH010). Hence, the BGCs found in the pathogenic isolates and their closest relatives were compared to determine differences in secondary metabolism of these microorganisms.

### Investigation of potential virulence factors in *Streptomyces *sp. JH002 and *Streptomyces* sp. JH010 genomes

Here we aimed to find genes that might be involved in the pathogenesis of these isolates by using two different approaches: (i) search for orthogroups of protein-coding genes unique in pathogenic *Streptomyces* species through Orthofinder, and (ii) identification of homologs of putative proteins involved in the synthesis of virulence factors commonly described in *Streptomyces* species through BlastP.

Orthofinder v2.4.0 with default parameters was used to obtain orthogroups for protein-coding genes from the genomes of pathogenic and non-pathogenic species [78]; specifically, it was used to find orthogroups from pathogenic species that are absent in non-pathogenic organisms. Subsequently, one protein sequence from each group was chosen randomly, and homologs were determined through BlastP (Version 2.11.0) search on NCBI under default parameters [79]. The *Streptomyces* species and accession numbers of genomes included in the Orthofinder analysis are shown in Table S5.

Sequences of proteins involved in the biosynthesis of virulence factors that have been described in pathogenic *Streptomyces* species were retrieved from the NCBI database and searched in the genome annotation of the pathogenic isolates using BlastP 2.5.0 with default parameters and E-value cut-off of 1e-4. Query cover, identity, and positive substitutions were obtained by BlastP on NCBI. Sequences from isolates with query cover ≥ 80% and identity ≥ 40% to protein sequences involved in virulence in pathogenic species were chosen for further analysis. Protein sequences chosen were analyzed with the HMMER 3.3.2 webserver to confirm their putative function [80]. Table S5 shows accession numbers for the protein sequences retrieved from the NCBI database.

Finally, Tat substrates homologous to proteins involved in the pathogenesis of phytopathogenic organisms were found. Putative proteins secreted through the Tat-system were predicted through TATFIND 1.4 [81] and TatP 1.0 servers [82] under default parameters. Only the proteins predicted by both servers were considered as *bona fide* Tat substrates. The function of *bona fide* Tat substrates was obtained from RAST annotation.

### Analysis of putative mART toxin encoded in the *Streptomyces* sp. JH002 genome

Analyses were conducted to confirm that a putative mono-ADP-ribosyltransferase (mART) toxin is encoded in the genome of the pathogenic isolate *Streptomyces* sp. JH002 by following the mART toxin discovery pipeline described by Tremblay et al., [36], with some modifications as follows: (i) we evaluated whether this protein has a similar folding to other mART toxins by analyzing the sequence in Local Meta-Threading Server (LOMETS). In addition, the putative protein 3D structure was predicted by using RaptorX template-based protein structure modeling under default parameters [83, 84]; (ii) the presence of secretion signal peptides or indicators of non-classical secretion and the lack of transmembrane domains in the sequences was determined by SignalP 5.0 and SecretomeP 2.0 with default parameters [85, 86]; (iii) conserved catalytic mART motifs from the sequence were identified through mART toxin sequence alignments conducted by MUSCLE; and (iv) the molecular function of the putative mART was predicted by using COFACTOR server under default parameters [87].

In addition, phylogenetic analysis based on protein sequences of mART and putative mART toxins was conducted. Accession numbers of protein sequences are shown in Table S6. The sequence alignment was conducted using MUSCLE in MEGA 7.0 and trimmed manually to the same position. The phylogenetic tree was carried out on Phylotree.fr by Mr. Bayes 3.2.6 with default parameters, except the substitution model implemented was Poisson and the number of generations was set to 100,000, parameters that yielded the highest Bayesian support values.

## Supplementary Information

**Additional file 1:** **Table S1.** Accession numbers or locus tag of the sequences retrieved from the NCBI database for MLSA. **Table S2.** Pairwise comparison results between 16S rRNA sequences from Streptomyces sp. JH34 and EZBioCloud database of the 30 species with similarity values ≥ 98.7%. **Table S3.** Pairwise comparison results between 16S rRNA sequences from Streptomyces sp. JH14 and EZBioCloud database of the 36 species with similarity values ≥ 98.7%. **Table S4.** Accession numbers of Streptomyces species assemblies used for ANI calculation. **Table S5.** Accession numbers of genome assemblies used in this study. **Table S6.** Putative biosynthetic gene clusters identified by antiSMASH from Streptomyces sp. JH002 genome. NA= known BGCs were not found **Table S7.** Putative biosynthetic gene clusters identified by antiSMASH from Streptomyces sp. JH010 genome. NA= known BGCs were not found. **Table S8.** Putative biosynthetic gene clusters identified by antiSMASH from Streptomyces sp. JH014 genome. NA= known BGCs were not found. **Table S9.** Putative biosynthetic gene clusters identified by antiSMASH from Streptomyces sp. JH34 genome. NA= known BGCs were not found. **Table S10.** Accession numbers of the protein sequences involved in the biosynthesis of virulence factors of pathogenic Streptomyces species. **Table S11.** Sequences identifiers of mART toxins included in the phylogenetic analysis. **Table S12.** Putative proteins secreted through Tat-system in Streptomyces sp. JH010 and Streptomyces sp. JH002 isolates. Tat substrates were predicted by TATFIND 1.4 and TatP 1.0 servers. **Figure S1.** Sequence alignment of piersin-like enzymes, scabin from Streptomyces scabiei, ScARP from Streptomyces coelicolor and the putative mART from Streptomyces sp. JH002 isolate. Residues conserved in key actives sites are indicated by red arrowheads. Image was generated in Snapgene software (from Insightful Science; available at snapgene.com).

## Data Availability

All genome assemblies and annotations are available at Genbank (see Table 1). Raw sequencing data has been submitted to NCBI with BioProject PRJNA657491. For review: https://dataview.ncbi.nlm.nih.gov/object/PRJNA657491?reviewer=fsl5ue96j8k542hcak925vs1js
