# Supplementary material for: Genomes of four Streptomyces strains reveal insights into putative new species and pathogenicity of scab-causing organisms
Source: BMC Genomics. 2023 Mar 23;24:143. doi: 10.1186/s12864-023-09190-y (PMC10037901; doi:10.1186/s12864-023-09190-y)
Supplement: Supplementary file 1 — Additional file 1: Table S1. Accession numbers or locus tag of the sequences retrieved from the NCBI database for MLSA. Table S2. Pairwise comparison results between 16S rRNA sequences from Streptomyces sp. JH34 and EZBioCloud database of the 30 species with similarity values ≥ 98.7%. Table S3. Pairwise comparison results between 16S rRNA sequences from Streptomyces sp. JH14 and EZBioCloud database of the 36 species with similarity values ≥ 98.7%. Table S4. Accession numbers of Streptomyces species assemblies used for ANI calculation. Table S5. Accession numbers of genome assemblies used in this study. Table S6. Putative biosynthetic gene clusters identified by antiSMASH from Streptomyces sp. JH002 genome. NA= known BGCs were not found Table S7. Putative biosynthetic gene clusters identified by antiSMASH from Streptomyces sp. JH010 genome. NA= known BGCs were not found. Table S8. Putative biosynthetic gene clusters identified by antiSMASH from Streptomyces sp. JH014 genome. NA= known BGCs were not found. Table S9. Putative biosynthetic gene clusters identified by antiSMASH from Streptomyces sp. JH34 genome. NA= known BGCs were not found. Table S10. Accession numbers of the protein sequences involved in the biosynthesis of virulence factors of pathogenic Streptomyces species. Table S11. Sequences identifiers of mART toxins included in the phylogenetic analysis. Table S12. Putative proteins secreted through Tat-system in Streptomyces sp. JH010 and Streptomyces sp. JH002 isolates. Tat substrates were predicted by TATFIND 1.4 and TatP 1.0 servers. Figure S1. Sequence alignment of piersin-like enzymes, scabin from Streptomyces scabiei, ScARP from Streptomyces coelicolor and the putative mART from Streptomyces sp. JH002 isolate. Residues conserved in key actives sites are indicated by red arrowheads. Image was generated in Snapgene software (from Insightful Science; available at snapgene.com). [file 12864_2023_9190_MOESM1_ESM.docx]

Supplemental Material: Genomes of four *Streptomyces* strains reveal insights into putative new species and pathogenicity of scab-causing organisms

Laura Henao^*^; Ramin Shirali Hossein Zade *^‡^*; Silvia Restrepo*^§^*; Johana Husserl^*^; Thomas Abeel *^‡,+,@^*

^*^Department of Civil and Environmental Engineering, Universidad de los Andes, Bogotá, Colombia, 111711;

^‡^ Department of Intelligent Systems, Delft University of Technology, Delft, Netherlands, 2628 XE;

^§^Laboratory of Mycology and Phytopathology - (LAMFU), Department of chemical and food engineering, Universidad de los Andes, Bogotá, Colombia, 111711;

^+^ Infectious Disease and Microbiome Program, Broad Institute of MIT and Harvard, 415 Main Street, Cambridge, MA, 02142, USA

^@^ Corresponding author: [t.abeel@tudelft.nl](mailto:t.abeel@tudelft.nl)

**Table S1.** Accession numbers or locus tag of the sequences retrieved from the NCBI database for MLSA

| **Name** | **Strain** | ***atpD*** | ***gyrB*** | ***recA*** | ***rpoB*** | ***trpB*** |
| --- | --- | --- | --- | --- | --- | --- |
| *Streptomyces yanii* | CGMCC 4.1146 | EF031275.1 | EF054964.1 | EF055017.1 | EF055072.1 | EF055127.1 |
| *Streptomyces xantholiticus* | NRRL B-12153 | KT384764.1 | KT385112.1 | KT385466.1 | KT389084.1 | KT389433.1 |
| *Streptomyces spiroverticillatus* | CGMCC 4.1749 | EF031304.1 | AB014337.1 | EF055046.1 | EF055101.1 | EF055156.1 |
| *Streptomyces sindenensis* | CGMCC 4.626 | EF031274.1 | EF054963.1 | EF055016.1 | EF055071.1 | EF055126.1 |
| *Streptomyces sanglieri* | CGMCC 41146 | EF661727.1 | EF661748.1 | EF661769.1 | EF661790.1 | EF661811.1 |
| *Streptomyces rubiginosohelvolus* | CGMCC 4.127 | EF661726.1 | EF661747.1 | EF661768.1 | EF661789.1 | EF661810.1 |
| *Streptomyces puniceus* | CGMCC 4.1750 | EF661724.1 | EF661745.1 | EF661766.1 | F661787.1 | EF661808.1 |
| *Streptomyces pulveraceus* | CGMCC 4.1928 | EF031323.1 | EF055010.1 | EF055065.1 | EF055120.1 | EF055175.1 |
| *Streptomyces pratensis* | ch24 | JQ806230.1 | JQ806234.1 | JQ806238.1 | JQ806242.1 | JQ806246.1 |
| *Streptomyces pluricolorescens* | CGMCC 4.236 | EF661723.1 | EF661744.1 | EF661765.1 | EF661786.1 | EF661807.1 |
| *Streptomyces parvus* | CGMCC 4.610 | EF661722.1 | EF661743.1 | EF661764.1 | EF661785.1 | EF661806.1 |
| *Streptomyces nitrosporeus* | CGMCC 4.1973 | EF661720.1 | EF661741.1 | EF661762.1 | EF661783.1 | EF661804.1 |
| *Streptomyces mutomycini* | NRRL B-65393 | BFF76_RS15375 | BFF76_RS26260 | BFF76_RS14990 | BFF76_RS05315 | BFF76_RS25080 |
| *Streptomyces microflavus* | NRRL B-1332 | KC965065.1 | KC965073.1 | KC965081.1 | KC965089.1 | KC965097.1 |
| *Streptomyces lunaelactis* | MM109 | KX503410.1 | KM207219.1 | KX503480.1 | KJ862819.1 | KX503690.1 |
| *Streptomyces laculatispora* | NRRL B-24909 | KT384780.1 | KT385128.1 | KT385482.1 | KT389100.1 | KT389449.1 |
| *Streptomyces halstedii* | CGMCC 4.1358 | EF661719.1 | EF661740.1 | EF661761.1 | EF661782.1 | EF661803.1 |
| *Streptomyces griseus subsp.*  *griseus* | NRRL B-2682 | KT384588.1 | KT384937.1 | KT385286.1 | KT388908.1 | KT389257.1 |
| *Streptomyces globisporus subsp.*  *globisporus* | NRRL B-2872 | KT384568.1 | KT384917.1 | KT385266.1 | KT388887.1 | KT389237.1 |
| *Streptomyces gelaticus* | CGMCC 4.1444 | EF661715.1 | EF661736.1 | EF661757.1 | EF661778.1 | EF661799.1 |
| *Streptomyces fulvorobeus* | NBRC 15897 | Sfulv_RS20225 | Sfulv_RS14285 | Sfulv_RS21790 | Sfulv_36200 | Sfulv_RS06300 |
| *Streptomyces flavovirens* | CGMCC 4.0575 | EF661714.1 | EF661735.1 | EF661756.1 | EF661777.1 | EF661798.1 |
| *Streptomyces finlayi* | CGMCC 4.1436 | EF031287.1 | EF054976.1 | EF055029.1 | EF055084.1 | EF055139.1 |
| *Streptomyces fimicarius* | NRRL ISP-5322 | KT384549.1 | KT384898.1 | KT385247.1 | KT388868.1 | KT389218.1 |
| *Streptomyces cyaneofuscatus* | CGMCC 4.1612 | EF031291.1 | EF054980.1 | EF055033.1 | EF055088.1 | EF055143.1 |
| *Streptomyces cremeus* | NRRL 3241 | KF433024.1 | KF433032.1 | KF433040.1 | KF433048.1 | KF433056.1 |
| *Streptomyces clavifer* | CGMCC 4.1604 | EF661712.1 | EF661733.1 | EF661754.1 | EF661775.1 | EF661796.1 |
| *Streptomyces cavourensis subsp.*  *cavourensis* | NRRL ISP-5300 | KT384504.1 | KT384853.1 | KT385201.1 | KT388823.1 | KT389173.1 |
| *Streptomyces candidus* | NRRL ISP-5151 | KF433023.1 | KF433031.1 | KF433039.1 | KJ996824.1 | KF433055.1 |
| *Streptomyces brevispora* | NRRL B-24910 | KT384781.1 | KT385129.1 | KT385483.1 | KT389101.1 | KT389450.1 |
| *Streptomyces badius* | CGMCC 4.1406 | EF031282.1 | EF054971.1 | EF055024.1 | EF055079.1 | EF055134.1 |
| *Streptomyces bacillaris* | CGMCC 4.1584 | EF661709.1 | EF661730.1 | EF661751.1 | EF661772.1 | EF661793.1 |
| *Streptomyces atroolivaceus* | CGMCC 4.1405 | EF031281.1 | EF054970.1 | EF055023.1 | EF055078.1 | EF055133.1 |
| *Streptomyces atratus* | CGMCC 4.1632 | EF661707.1 | EF661728.1 | EF661749.1 | EF661770.1 | EF661791.1 |
| *Streptomyces anulatus* | NRRL B-2873 | KT384557.1 | KT384906.1 | KT385255.1 | KT388876.1 | KT389226.1 |
| *Kitasatospora papulosa'* | NRRL B-16504 | IA22_RS0112265 | IA22_RS0107280 | IA22_RS0124835 | KJ996383.1 | IA22_RS0104725 |
| *Streptomyces xiamenensis* | 318 | SXIM_RS20520 | SXIM_RS13310 | SXIM_RS22360 | SXIM_RS10370 | SXIM_RS04745 |
| *Streptomyces* sp*.* JH14 |  | LRD69_22700 | LRD69_16020 | LRD69_24660 | LRD69_19355 | LRD69_06915 |
| *Streptomyces* sp*.* JH34 |  | LWJ43_09850 | LWJ43_16300 | LWJ43_07895 | LWJ43_13115 | LWJ43_25105 |
| *Streptomyces* sp*.* JH010 |  | H8R03_22540 | H8R03_16560 | H8R03_24460 | H8R03_19660 | H8R03_08290 |
| *Streptomyces* sp. JH002 |  | H7827_07275 | H7827_14745 | H7827_05395 | H7827_17865 | H7827_23850 |

Table S2. Pairwise comparison results between 16S rRNA sequences from *Streptomyces* sp. JH34 and EZBioCloud database of the 30 species with similarity values ≥ 98.7%.

| **Name** | **Strain** | **Accession** | **Pairwise Similarity (%)** |
| --- | --- | --- | --- |
| *Streptomyces clavifer* | NRRL B-2557 | DQ026670 | 100 |
| *Streptomyces mutomycini* | NRRL B-65393 | MAPV01000102 | 100 |
| *Streptomyces atroolivaceus* | NRRL ISP-5137 | JNXG01000049 | 99.93093923 |
| *Streptomyces finlayi* | NRRL B-12114 | AY999788 | 99.79281768 |
| *Streptomyces setonii* | NRRL ISP-5322 | MUNB01000146 | 99.44751381 |
| *Streptomyces anulatus* | NRRL B-2000 | DQ026637 | 99.44751381 |
| *Kitasatospora papulosa* | NRRL B-16504 | JNYQ01000038 | 99.44751381 |
| *Streptomyces pratensis* | ch24 | JQ806215 | 99.41605839 |
| *Streptomyces badius* | NRRL B-2567 | AY999783 | 99.37845304 |
| *Streptomyces globisporus* | NBRC 12867 | AB184203 | 99.37759336 |
| *Streptomyces sindenensis* | NBRC 3399 | AB184759 | 99.37759336 |
| *Streptomyces parvus* | NBRC 3388 | AB184756 | 99.37759336 |
| *Streptomyces pluricolorescens* | NBRC 12808 | AB184162 | 99.37629938 |
| *Streptomyces rubiginosohelvolus* | NBRC 12912 | AB184240 | 99.375 |
| *Streptomyces cyaneofuscatus* | NRRL B-2570 | JOEM01000050 | 99.30939227 |
| *Streptomyces microflavus* | NBRC 13062 | AB184284 | 99.308915 |
| *Streptomyces puniceus* | NBRC 12811 | AB184163 | 99.308915 |
| *Streptomyces griseus* subsp. *griseus* | KCTC 9080 | M76388 | 99.30843707 |
| *Streptomyces fulvorobeus* | NBRC 15897 | AB184711 | 99.30795848 |
| *Streptomyces flavovirens* | NBRC 3716 | AB184834 | 99.17127072 |
| *Streptomyces halstedii* | NBRC 12783 | AB184142 | 99.170698 |
| *Streptomyces bacillaris* | NBRC 13487 | AB184439 | 99.1015895 |
| *Streptomyces pulveraceus* | LMG 20322 | AJ781377 | 98.9640884 |
| *Streptomyces candidus* | NRRL ISP-5141 | DQ026663 | 98.96337249 |
| *Streptomyces sundarbansensis* | MS1/7 | AY550275 | 98.96049896 |
| *Streptomyces nitrosporeus* | NBRC 3362 | AB184751 | 98.89273356 |
| *Streptomyces yanii* | NBRC 14669 | AB006159 | 98.84057971 |
| *Streptomyces cavourensis* | NBRC 13026 | AB184264 | 98.82515549 |
| *Streptomyces sanglieri* | NBRC 100784 | AB249945 | 98.75690608 |
| *Streptomyces lunaelactis* | MM109 | KM207217 | 98.73239437 |

Table S3 Pairwise comparison results between 16S rRNA sequences from *Streptomyces* sp. JH14 and EZBioCloud database of the 36 species with similarity values ≥ 98.7%.

| **Name** | **Strain** | **Accession** | **Pairwise Similarity (%)** | |
| --- | --- | --- | --- | --- |
| *Streptomyces yanii* | NBRC 14669 | AB006159 | | 99.92753623 |
| *Streptomyces atratus* | NRRL B-16927 | DQ026638 | | 99.86187845 |
| *Streptomyces sanglieri* | NBRC 100784 | AB249945 | | 99.79281768 |
| *Streptomyces pulveraceus* | LMG 20322 | AJ781377 | | 99.58563536 |
| *Streptomyces gelaticus* | NRRL B-2928 | DQ026636 | | 99.44751381 |
| *Streptomyces badius* | NRRL B-2567 | AY999783 | | 99.44751381 |
| *Streptomyces globisporus* | NBRC 12867 | AB184203 | | 99.44674965 |
| *Streptomyces sindenensis* | NBRC 3399 | AB184759 | | 99.44674965 |
| *Streptomyces parvus* | NBRC 3388 | AB184756 | | 99.44674965 |
| *Streptomyces pluricolorescens* | NBRC 12808 | AB184162 | | 99.44559945 |
| *Streptomyces rubiginosohelvolus* | NBRC 12912 | AB184240 | | 99.44444444 |
| *Streptomyces setonii* | NRRL ISP-5322 | MUNB01000146 | | 99.37845304 |
| *Streptomyces anulatus* | NRRL B-2000 | DQ026637 | | 99.37845304 |
| *Kitasatospora papulosa* | NRRL B-16504 | JNYQ01000038 | | 99.37845304 |
| *Streptomyces griseus* subsp*. griseus* | KCTC 9080 | M76388 | | 99.37759336 |
| *Streptomyces pratensis* | ch24 | JQ806215 | | 99.34306569 |
| *Streptomyces flavovirens* | NBRC 3716 | AB184834 | | 99.24033149 |
| *Streptomyces cyaneofuscatus* | NRRL B-2570 | JOEM01000050 | | 99.24033149 |
| *Streptomyces microflavus* | NBRC 13062 | AB184284 | | 99.2398065 |
| *Streptomyces halstedii* | NBRC 12783 | AB184142 | | 99.2398065 |
| *Streptomyces puniceus* | NBRC 12811 | AB184163 | | 99.2398065 |
| *Streptomyces fulvorobeus* | NBRC 15897 | AB184711 | | 99.23875433 |
| *Streptomyces nitrosporeus* | NBRC 3362 | AB184751 | | 99.10034602 |
| *Streptomyces bacillaris* | NBRC 13487 | AB184439 | | 99.032481 |
| *Streptomyces brevispora* | BK160 | FR692104 | | 99.0237099 |
| *Streptomyces lunaelactis* | MM109 | KM207217 | | 98.94366197 |
| *Streptomyces atroolivaceus* | NRRL ISP-5137 | JNXG01000049 | | 98.89502762 |
| *Streptomyces finlayi* | NRRL B-12114 | AY999788 | | 98.89502762 |
| *Streptomyces sundarbansensis* | MS1/7 | AY550275 | | 98.89119889 |
| *Streptomyces cremeus* | NBRC 12760 | AB184124 | | 98.88811675 |
| *Streptomyces laculatispora* | BK166 | FR692106 | | 98.88423989 |
| *Streptomyces clavifer* | NRRL B-2557 | DQ026670 | | 98.82596685 |
| *Streptomyces spiroverticillatus* | NBRC 12821 | AB249921 | | 98.82596685 |
| *Streptomyces mutomycini* | NRRL B-65393 | MAPV01000102 | | 98.82596685 |
| *Pilimelia columellifera* subsp*. pallida* | MB-SK 8 | GU269552 | | 98.74651811 |
| *Streptomyces xantholiticus* | NBRC 13354 | AB184349 | | 98.74476987 |

**Table S4.** Accession numbers of *Streptomyce*s species assemblies used for ANI calculation.

| **Name** | **Accession Number** |
| --- | --- |
| *Streptomyces pratensis* ATCC 33331 | GCA_000176115.2 |
| *‘Kitasatospora papulosa’* NRRL B-16504 | GCA_000717245.1 |
| *Streptomyces clavifer* CGMCC 4.1604 | GCA_017876255.1 |
| *Streptomyces atroolivaceus* CGMCC 4.1405 | GCA_000717025.1 |
| *Streptomyces mutomycini* NRRL B-65393 | GCA_001700505.1 |
| *Streptomyces laculatispora* NRRL B-24909 | GCA_017353455.1 |

| **Reference isolate/strain** | **Analyses** | **Accession number** |
| --- | --- | --- |
| *Streptomyces scabiei* 87-22 | Phylogenomic Analysis- Pangenome | NC_013929.1 |
| *Streptomyces europaeiscabiei* 96-14 | Phylogenomic Analysis- Pangenome | GCA_001550375.1 |
| *Streptomyces turgidiscabies* Car8 | Phylogenomic Analysis- Pangenome | GCA_000331005.1 |
| *Streptomyces acidiscabies* NRRL B-16524 | Phylogenomic Analysis | GCA_002150705.1 |
| *Streptomyces acidiscabies* 84-104 | Phylogenomic Analysis- Pangenome | GCA_000242715.2 |
| *Streptomyces niveiscabiei* NRRL B-24457 | Phylogenomic Analysis- Pangenome | GCA_001419795.1 |
| *Streptomyces luridiscabiei* NRRL B-24455 | Phylogenomic Analysis-Pangenome | GCA_001418625.1 |
| *Streptomyces puniciscabiei* DSM41929 | Phylogenomic Analysis | GCA_006715785.1 |
| *Streptomyces reticuliscabiei* NRRL B-24446 | Phylogenomic Analysis-Pangenome | GCA_002154675.1 |
| *Streptomyces stelliscabiei* P3825 | Phylogenomic Analysis- Pangenome | GCA_001189035.1 |
| *Streptomyces ipomoeae* 91-03 | Phylogenomic Analysis- Pangenome | GCA_000317595.1 |
| *Streptomyces* sp. ST1015 | Phylogenomic Analysis- Pangenome | GCA_003268535.2 |
| *Streptomyces* sp. ST1020 | Phylogenomic Analysis- Pangenome | GCA_003268555.1 |
| *Streptomyces pratensis* ATCC 33331 | Phylogenomic Analysis- Pangenome | GCA_000176115.2 |
| *Streptomyces xiamenensis* 318 | Phylogenomic Analysis | GCA_000993785.3 |
| *Norcadopsis assonvillei* NCTC 10488 | Phylogenomic Analysis | GCA_900638215.1 |
| *Streptomyces avermitilis* MA4680 | Pangenome | GCA_000009765.2 |
| *Streptomyces bingchenggensis* BCW1 | Pangenome | NC_016582.1 |
| *Streptomyces cattleya* DMS46488 | Pangenome | GCA_000240165.1 |
| *Streptomyces coelicolor* A3 | Pangenome | GCA_008931305.1 |
| *Streptomyces griseus* subsp. *griseus* NBRC 13350 | Pangenome | GCA_000010605.1 |
| *Streptomyces hygroscopicus* subsp. *jinggangensis* 5008 | Pangenome | NC_017765.1 |
| *Streptomyces* sp. SirexAA-E | Pangenome | NC_015953.1 |
| *Streptomyces venezuelae* ATCC 10712 | Pangenome | NZ_CP029197.1 |
| *Streptomyces violaceusniger* Tu 4113 | Pangenome | NC_015957.1 |

**Table S5**. Accession numbers of genome assemblies used in this study.

Table S6. Putative biosynthetic gene clusters identified by antiSMASH from *Streptomyces sp.* JH002 genome. NA= known BGCs were not found

| Number | Type | Most similar known cluster | | Similarity |
| --- | --- | --- | --- | --- |
|  |  | **Name** | **Type** |  |
| 1 | NRPS | s56-p1 | NRP | 15% |
| 2 | T1PKS | butyrolactol A | Polyketide | 26% |
| 3 | NRPS | WS9326 | NRP | 15% |
| 4 | NRPS,T1PKS | foxicins A-D | NRP + Polyketide | 4% |
| 5 | NRPS,terpene | spiroindimicin A / spiroindimicin B / spiroindimicin C / spiroindimicin D / indimicin A / indimicin B / indimicin C / indimicin D / indimicin E / lynamicin A / lynamicin D / lynamicin F / lynamicin G | Other | 21% |
| 6 | T3PKS | herboxidiene | Polyketide | 2% |
| 7 | terpene | hopene | Terpene | 30% |
| 8 | lassopeptide | moomysin | RiPP | 75% |
| 9 | NRPS,T1PKS | carotenoid | Terpene | 18% |
| 10 | NRPS | valinomycin / montanastatin | NRP + Saccharide:Hybrid/tailoring | 17% |
| 11 | siderophore | NA | NA | NA |
| 12 | thiopeptide,LAP | conglobatin | NRP | 10% |
| 13 | terpene | NA | NA | NA |
| 14 | T1PKS,NRPS,linaridin | ikarugamycin | NRP + Polyketide:Iterative type I | 84% |
| 15 | linaridin,lanthipeptide | NA | NA | NA |
| 16 | T2PKS | nenestatin | Polyketide | 66% |
| 17 | butyrolactone | methylenomycin A | Other | 9% |
| 18 | T1PKS,NRPS | NA | NA | NA |
| 19 | butyrolactone | NA | NA |  |
| 20 | terpene | chlortetracycline | Polyketide | 5% |
| 21 | nucleoside |  | NA | NA |
| 22 | ectoine | ectoine | Other | 100% |
| 23 | siderophore | desferrioxamin B | Other | 60% |

Table S7. Putative biosynthetic gene clusters identified by antiSMASH from *Streptomyces* sp. JH010 genome. NA= known BGCs were not found

| Number | Type | Most similar known cluster | | Similarity |
| --- | --- | --- | --- | --- |
|  |  | **Name** | **Type** |  |
| 1 | butyrolactone | NA | NA |  |
| 2 | NRPS | rimosamide | NRP | 21% |
| 3 | T2PKS,terpene | spore pigment | Polyketide | 83% |
| 4 | melanin | melanin | Other | 100% |
| 5 | T3PKS | tetronasin | Polyketide | 11% |
| 6 | bacteriocin | NA | NA |  |
| 7 | terpene,ectoine | ectoine | Other | 100% |
| 8 | terpene | steffimycin D | Polyketide:Type II + Saccharide:Hybrid/tailoring | 16% |
| 9 | T2PKS | cinerubin B | Polyketide:Type II | 25% |
| 10 | ectoine | ectoine | Other | 100% |
| 11 | terpene | NA | NA |  |
| 12 | lanthipeptide | azalomycin F3a | Polyketide | 8% |
| 13 | siderophore | desferrioxamin B / desferrioxamine E | Other | 83% |
| 14 | NRPS,T1PKS | istamycin | Saccharide | 11% |
| 15 | butyrolactone | lactonamycin | Polyketide | 3% |
| 16 | bacteriocin | NA | NA |  |
| 17 | terpene | NA | NA |  |
| 18 | siderophore | ficellomycin | NRP | 3% |
| 19 | NRPS | cadaside A / cadaside B | NRP | 19% |
| 20 | bacteriocin | NA | NA |  |
| 21 | T1PKS | sceliphrolactam | Polyketide | 88% |
| 22 | terpene | hopene | Terpene | 69% |
| 23 | blactam | clavulanic acid | Other:Non-NRP beta-lactam | 20% |
| 24 | bacteriocin | NA | NA |  |
| 25 | terpene | isorenieratene | Terpene | 100% |
| 26 | NRPS | coelichelin | NRP | 90% |
| 27 | NRPS,blactam,T1PKS | carbapenem MM4550 | Other:Non-NRP beta-lactam | 65% |

Table S8. Putative biosynthetic gene clusters identified by antiSMASH from *Streptomyces* sp. JH014 genome. NA= known BGCs were not found.

| **Number** | **Type** | | | **Most similar known cluster** | | **Similarity** |
| --- | --- | --- | --- | --- | --- | --- |
|  |  |  |  | **Name** | **Type** |  |
| 1 | bacteriocin | | | desotamide | NRP | 9% |
| 2 | terpene | | | steffimycin D | Polyketide:Type II + Saccharide:Hybrid/tailoring | 19% |
| 3 | butyrolactone | | NA | | NA | NA |
| 4 | siderophore | | | desferrioxamin B | Other | 100% |
| 5 | T3PKS | | | naringenin | Terpene | 100% |
| 6 | phosphonate,T1PKS,ladderane | | | amycomicin | Polyketide | 100% |
| 7 | terpene | | | BD-12 | NRP | 17% |
| 8 | siderophore | NA | | | NA | NA |
| 9 | bacteriocin | | | |  |  |
| 10 | NRPS | | | s56-p1 | NRP | 5% |
| 11 | terpene | | | hopene | Terpene | 84% |

Table S9. Putative biosynthetic gene clusters identified by antiSMASH from *Streptomyces* sp. JH34 genome. NA= known BGCs were not found.

| **Number** | **Type** | **Most similar known cluster** | | **Similarity** |
| --- | --- | --- | --- | --- |
|  |  | **Name** | |  |
| 1 | terpene | xiamycin | Terpene + Alkaloid | 20% |
| 2 | NRPS | rimosamide | NRP | 21% |
| 3 | NRPS | coelichelin | NRP | 90% |
| 4 | terpene | isorenieratene | Terpene | 100% |
| 5 | bacteriocin | olimycin A / olimycin B | Polyketide | 5% |
| 6 | T2PKS,oligosaccharide | chromomycin A3 | Polyketide:Type II + Saccharide:Oligosaccharide | 88% |
| 7 | blactam | clavulanic acid | Other:Non-NRP beta-lactam | 20% |
| 8 | terpene | hopene | Terpene | 76% |
| 9 | T1PKS | sceliphrolactam | Polyketide | 92% |
| 10 | bacteriocin | NA | NA | NA |
| 11 | NRPS | cadaside A / cadaside B | NRP | 19% |
| 12 | siderophore | ficellomycin | NRP | 3% |
| 13 | terpene | NA | NA | NA |
| 14 | bacteriocin | NA | NA | NA |
| 15 | butyrolactone | lactonamycin | Polyketide | 3% |
| 16 | lanthipeptide | NA | NA | NA |
| 17 | siderophore | desferrioxamin B / desferrioxamine E | Other | 100% |
| 18 | lanthipeptide | azalomycin F3a | Polyketide | 8% |
| 19 | terpene | NA | NA | NA |
| 20 | ectoine | ectoine | Other | 100% |
| 21 | oligosaccharide | glycinocin A | NRP | 9% |
| 22 | terpene | steffimycin D | Polyketide:Type II + Saccharide:Hybrid/tailoring | 16% |
| 23 | terpene,ectoine,hglE-KS,T1PKS | ectoine | Other | 100% |
| 24 | bacteriocin | NA | NA | NA |
| 25 | T3PKS | tetronasin | Polyketide | 11% |
| 26 | melanin | melanin | Other | 100% |
| 27 | T2PKS,terpene | spore pigment | Polyketide | 83% |

| **Protein** | **Organism** | **Gene** | **Accession Number** |
| --- | --- | --- | --- |
| **Thaxtomin** |  |  |  |
| Thaxtomin synthetase A | *Streptomyces acidiscabies* | *txtA* | AAG27087.1 |
| Thaxtomin synthetase B | *Streptomyces acidiscabies* | *txtB* | AAG27088.1 |
| **Coronafacoyl phytotoxins** |  |  |  |
| acyl--CoA ligase | *Streptomyces scabiei* | *cfl* | WP_013005377.1 |
| **Concanamycin A** |  |  |  |
| Modular polyketide synthase | *Streptomyces neyagawaensis* | *conE* | AAZ94390.1 |
| **18-membered macrolide borrelidin** |  |  |  |
| Borrelidin polyketide synthase, type I | *Streptomyces parvulus* | *borA3* | CAE45669.1 |
| **FD-891** |  |  |  |
| polyketide synthase | *Streptomyces graminofaciens* | *gfsA* | BAJ16467.2 |
| **Cytokinins** |  |  |  |
| cytochrome P450 | *Streptomyces turgidiscabies* Car8 | *fas1* | AAW49308.1 |
| putative ferredoxin transketolase |  | *fas2* | AAW49307.1 |
| hypothetical transketolase |  | *fas3* | AAW49306.1 |
| isopentyl transferase |  | *fas4* | AAW49305.1 |
| cytokinin oxidase |  | *fas5* | AAW49304.1 |
| Hypothetical protein stPAI019 |  | *fas6* | AAW49312.1 |
| **Indole-3-acetic acid** |  |  |  |
| FAD-dependent oxidoreductase | *Streptomyces scabiei* | *scab_75511* homologous of *iaaM* | WP_013005061.1 |
| carbon-nitrogen hydrolase family protein | *Streptomyces scabiei* | *scab_75501* homologous of *iaaH* | WP_013005060.1 |
| **Ethylene** |  |  |  |
| 2-oxoglutarate-dependent ethylene/succinate-forming enzyme | *Streptomyces scabiei* | *efe_1* | GAQ60554.1 |
| **Proteins** |  |  |  |
| Necrogenic protein Nec1 | *Streptomyces* sp. 87.76 | *nec1* | AAL76207.1 |
| Putative Tomatinase | *Streptomyces scabiei* | *tomA* | CBG74701.1 |
| Scabin | *Streptomyces scabiei* | *scab_RS13165* | WP_037722833.1 |
| Putative suberinase | *Streptomyces scabiei* | *estA* | AAA26744.1 |
| Putative suberinase | *Streptomyces scabiei* | *sub1* | WP_013005307.1 |
| Lipoprotein | *Streptomyces scabiei* | *scab _76661* | WP_013005169.1 |
| Putative cellulase | *Streptomyces scabiei* | *scab _90061* | CBG75940.1 |
| Pathogenesis-related protein-like | *Streptomyces scabiei* | *scab _44951* | CBG71558.1 |
| **Tat-system** |  |  |  |
| TatA | *Streptomyces scabiei* | *tatA* | WP_164326297.1 |
| TatB | *Streptomyces scabiei* | *tatB* | WP_019434899.1 |
| TatC | *Streptomyces scabiei* | *tatC* | WP_013004885.1 |

**Table S10**. Accession numbers of the protein sequences involved in the biosynthesis of virulence factors of pathogenic *Streptomyces* species.

**Table S11.** Sequences identifiers of mART toxins included in the phylogenetic analysis.

| **Protein Identifier from UniProtKB/PDB** | **Name** | **Organism** |
| --- | --- | --- |
| A0A1W5T8P6 | Edin A | *Staphylococcus aureus* |
| Q8GAX6 | Edin B | *Staphylococcus aureus* |
| 2C89 | C3bot1 | *Clostridium botulinum* |
| E5B8T9 | Vorin | *Erwinia amylovora* |
| Q00901 | C3bot2 | *Clostridium botulinum C phage* |
| P75409 | CARDS | *Mycoplasma pneumoniae* |
| K0MBH6 | PT | *Bordetella parapertussis* |
| E7EKM3 | Pierisin-1b | *Pieris rapae* |
| H3JU00 | Pierisin-1 | *Pieris rapae* |
| Q9GV36 | Pierisin | *Pieris brassicae* |
| C6L2F5 | Pierisin-3 | *Pieris melete* |
| C9Z6T8 | Scabin | *Streptomyces scabiei* |
| 5ZJ4 | ScARP | *Streptomyces coelicolor* |
| A0A6B3Q9D9 | VopT | *Vibrio parahaemolyticus* |
| Q51451 | ExoS | *Pseudomonas aeruginosa* |
| D4N871 | C2I | *Clostridium botulinum* |
| O06497 | Sa | *Clostridium spiroforme* |

**Table S12.** Putative proteins secreted through Tat-system in *Streptomyces* sp*.* JH010 and *Streptomyces* sp*.* JH002 isolates. Tat substrates were predicted by TATFIND 1.4 and TatP 1.0 servers.

| ***Streptomyces* sp*.* JH010** | ***Streptomyces* sp*.* JH002** |
| --- | --- |
| FIG01121776: hypothetical protein | Putative secreted protein Serine Protease (PDB) |
| putative cholesterol oxidase | Putative secreted protein Glycosylhydrolase |
| hypothetical protein | Putative major teichoic acid biosynthesis protein C |
| Predicted rhamnose oligosaccharide ABC transport system, substrate-binding component | Oligopeptide ABC transporter, periplasmic oligopeptide-binding protein OppA (TC 3.A.1.5.1) |
| FIG01129755: hypothetical protein | Amino acid ABC transporter, periplasmic amino acid-binding protein |
| Glycerophosphoryl diester phosphodiesterase (EC 3.1.4.46) | Lon-like protease with PDZ domain |
| alternate gene name: yzbB | Hypothetical protein; putative N-acetylmuramoyl-L-alanine amidase domains |
| phosphodiesterase/alkaline phosphatase D | 2',3'-cyclic-nucleotide 2'-phosphodiesterase (EC 3.1.4.16) |
| putative secreted pectinesterase | Putative NLP/P60-family protein |
| putative iron sulphur protein | Putative esterase |
| 2',3'-cyclic-nucleotide 2'-phosphodiesterase (EC 3.1.4.16) | FIG01121207: hypothetical protein |
| putative secreted alkaline phosphatase | FIG00761799: membrane protein |
| Ferrous iron transport peroxidase EfeB | Hypothetical protein |
| Putative aldo/keto reductase | Predicted dye-decolorizing peroxidase (DyP), YfeX-like subgroup |
| Aldose 1-epimerase (EC 5.1.3.3) | Putative membrane protein |
| FIG01123273: hypothetical protein | Iron sulphur protein (secreted protein) |
| FIG00816166: hypothetical protein | Sugar phosphate isomerases/epimerases |
| Beta-hexosaminidase (EC 3.2.1.52) | Hypothetical protein |
| FIG01128882: hypothetical protein | Endo-1,4-beta-xylanase A precursor (EC 3.2.1.8) |
| Putative Desferrioxamine E transporter | Putative lipoprotein precursor lplA |
| putative sugar binding secreted protein | Predicted rhamnogalacturonan lyase in rhamnose utilization cluster |
| Phospholipase C | Putative integral membrane protein |
| Predicted dye-decolorizing peroxidase (DyP), YfeX-like subgroup | Hypothetical protein |
| Phosphatidylinositol-specific phospholipase C (EC 4.6.1.13) | Hypothetical protein |
| FIG01129122: hypothetical protein | ComEC/Rec2-related protein |
| FIG01121945: hypothetical protein | TctC citrate transporter |
| Endo-1,4-beta-xylanase | Putative dioxygenase |
| secreted endo-1,4-beta-xylanase B (xylanase B) | FIG00665196: hypothetical protein |
| FIG01133939: hypothetical protein | Putative galactosidase |
| sugar ABC transporter sugar-binding protein | FIG01131534: hypothetical protein |
| N-acetylmuramoyl-L-alanine amidase (EC 3.5.1.28) | Secreted protein |
| ABC-type Fe3+-hydroxamate transport system, periplasmic component | Glycerophosphoryl diester phosphodiesterase (EC 3.1.4.46) |
| FIG01128511: hypothetical protein | FIG01121776: hypothetical protein |
| Alfa-L-rhamnosidase (EC 3.2.1.40) | Aldose 1-epimerase (EC 5.1.3.3) |
| Putative glycosyl hydrolase of unknown function (DUF1680) | Hydroxymethylpyrimidine ABC transporter, substrate-binding component |
| Beta-galactosidase (EC 3.2.1.23) | Hypothetical protein |
| Inositol transport system sugar-binding protein | Chitinase (EC 3.2.1.14) |
| FIG01133064: hypothetical protein | Alpha-L-arabinofuranosidase II precursor (EC 3.2.1.55) |
| FIG01121207: hypothetical protein | FIG01132164: hypothetical protein |
| Cytosine deaminase (EC 3.5.4.1) | FIG01130718: hypothetical protein |
| FIG01132295: hypothetical protein | Sugar ABC transporter sugar-binding protein |
| ABC-type dipeptide transport system, periplasmic component | Peptidase S8 and S53, subtilisin, kexin, sedolisin |
| FIG01132164: hypothetical protein |  |
| Ribose ABC transporter, periplasmic ribose-binding protein RbsB (TC 3.A.1.2.1) |  |
| FIG01124897: hypothetical protein |  |
| secreted cellulase |  |
| FIG01123265: hypothetical protein |  |
| secreted protein Metallo beta-lactamase fold protein (cAMP free) PDB |  |
| FIG01132164: hypothetical protein |  |
| peptide ABC transporter peptide-binding protein,putative |  |
| Beta-galactosidase (EC 3.2.1.23) |  |
| DNA for SgaA, complete cds |  |
| putative secreted protein |  |
| N-acetylmuramoyl-L-alanine amidase (EC 3.5.1.28) |  |
| rhamnogalacturonan acetylesterase |  |
| secreted protein |  |
| putative glycosyl hydrolase |  |
| secreted protein |  |
| putative secreted alkaline phosphatase |  |
| Iron sulphur protein |  |

**Figure S1.** Sequence alignment of piersin-like enzymes, scabin from *Streptomyces scabiei*, ScARP from *Streptomyces coelicolor* and the putative mART from *Streptomyces* sp. JH002 isolate. Residues conserved in key actives sites are indicated by red arrowheads. Image was generated in Snapgene software (from Insightful Science; available at snapgene.com).
